# Neural networks versus Logistic regression for 30 days all-cause readmission prediction

**DOI:** 10.1038/s41598-019-45685-z

**Published:** 2019-06-26

**Authors:** Ahmed Allam, Mate Nagy, George Thoma, Michael Krauthammer

**Affiliations:** 1 Chair of Medical Informatics, Department of Quantitative Biomedicine, University of Zurich, Zurich, Switzerland; 2 Biomedical Informatics, University Hospital of Zurich, Zurich, Switzerland; 3 Program in Computational Biology and Bioinformatics, Yale University School of Medicine, New Haven, Connecticut USA; 4 Department of Pathology, Yale University School of Medicine, New Haven, Connecticut USA; 5 Lister Hill National Center for Biomedical Communications, National Library of Medicine, Bethesda, Maryland USA

**Keywords:** Outcomes research, Health care, Prognosis, Computer science, Statistics

## Abstract

Heart failure (HF) is one of the leading causes of hospital admissions in the US. Readmission within 30 days after a HF hospitalization is both a recognized indicator for disease progression and a source of considerable financial burden to the healthcare system. Consequently, the identification of patients at risk for readmission is a key step in improving disease management and patient outcome. In this work, we used a large administrative claims dataset to (1) explore the systematic application of neural network-based models versus logistic regression for predicting 30 days all-cause readmission after discharge from a HF admission, and (2) to examine the additive value of patients’ hospitalization timelines on prediction performance. Based on data from 272,778 (49% female) patients with a mean (SD) age of 73 years (14) and 343,328 HF admissions (67% of total admissions), we trained and tested our predictive readmission models following a stratified 5-fold cross-validation scheme. Among the deep learning approaches, a recurrent neural network (RNN) combined with conditional random fields (CRF) model (RNNCRF) achieved the best performance in readmission prediction with 0.642 AUC (95% CI, 0.640–0.645). Other models, such as those based on RNN, convolutional neural networks and CRF alone had lower performance, with a non-timeline based model (MLP) performing worst. A competitive model based on logistic regression with LASSO achieved a performance of 0.643 AUC (95% CI, 0.640–0.646). We conclude that data from patient timelines improve 30 day readmission prediction, that a logistic regression with LASSO has equal performance to the best neural network model and that the use of administrative data result in competitive performance compared to published approaches based on richer clinical datasets.

## Introduction

Heart failure (HF) is one of the leading causes for hospital admissions in the US^1–4^ with high numbers of readmissions within 30 days of discharge^2–4^. Based on multiple hospitalization data sources, the yearly rate of 30 days all-cause readmission after an HF hospitalization is approximately 23–24%^1,2,5^, posing a huge burden on the healthcare system with an estimated cost of $17 billions of total Medicare expenditure^4,6^. Beyond the associated expenses and costs, readmissions have negative consequences on patients’ health status, leading to complications and increased risk of disease progression^6^. Efforts toward quality improvement such as introducing programs that incentivize and penalize hospitals based on the yearly readmission rate have been the focus of researchers and policy makers^2,7^. Likewise, there has been an increasing interest in developing predictive models and/or monitoring systems that allow for prevention and preemptive steps^8,9^, such as the prediction of 30 days all-cause readmission for patients hospitalized with HF for which many challenges remain^10,11^.

In this paper, we aim at exploring the systematic application of neural network models for predicting 30 days all-cause readmission after discharge from a HF hospitalization (which we call index event below). Concretely, given a set of sequences of hospitalization admissions with their corresponding 30 days all-cause readmission outcome, we seek to predict the 30 days all-cause readmission of the last HF admission (i.e. the last index event) in each sequence. The sequence of hospitalization events for each patient will be referred to as “*timeline*” and “*trajectory*” interchangeably throughout the paper. Published approaches chiefly use data from the index event for predicting hospital readmission, paying less attention to a patient’s trajectory leading to the current heart failure admission. Intuitively, a patient’s history may add much additional information that may be informative of whether a patient is subject to early readmission. For example, a history of multiple readmissions in the past may be a risk factor for future readmissions. Consequently, one specific aim of this study is to examine the value of including a patient’s trajectory data in a 30 day readmission prediction model. To this end, we examine three approaches for modeling the problem of which two use the temporal information encoded in the patients’ trajectories (sequence labeling and sequence classification), and one that does not (index event classification). Particularly, we implemented multiple neural network models with varying architectures and objective functions such as recurrent neural networks (RNN), and convolutional neural networks (CNN) as examples of sequence labeling and classification approaches, and multilayer perceptron (MLP) along with logistic regression as baseline models representing the index event classification approach. We conducted these studies with a large administrative claims dataset, which lacks the detailed clinical information found in datasets typically used for this problem. As claims data are readily available and can be robustly harmonized, they pose less privacy concerns and are ideally suited for tacking the HF readmission problem.

## Results

**Figure 1:**
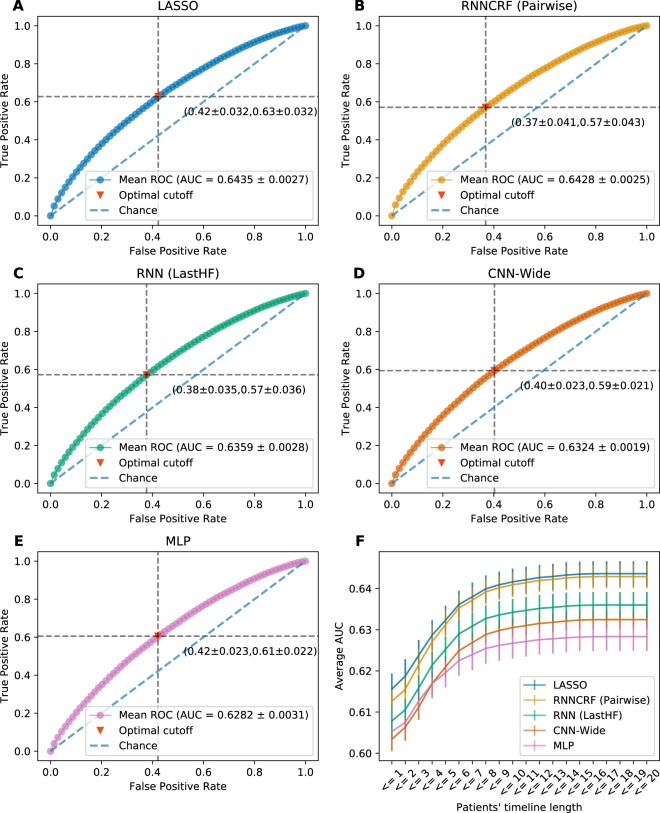
Performance analysis of the tested models. Panels A–E report the average ROC curve of the best models. The optimal cutoff  is based on the average Youden-Index of each model for all 5-folds. Standard deviation of the optimal cutoff position is reported on the graph. Panel F reports the cumulative average AUC performance as a function of patients’ timeline length.

**Figure 2:**
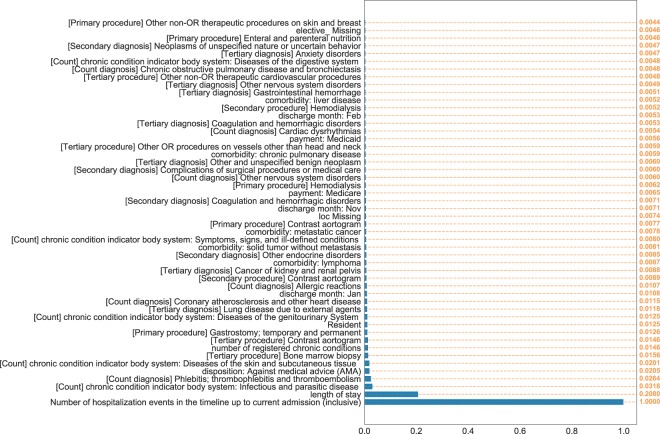
Top-50 features in LASSO models contributing to the increase of log-odds of readmission. The coefficients were normalized using the maximum absolute value of the models’ trained weights.

The HF dataset included 272,778 patients (49% female) with a mean (SD) age of 72.89 years (14). The total number of HF admissions was 343,328 (66.9% of all admissions) of which 81,087 (23.6%) were 30 days all-cause readmissions, corresponding to the official rates published by HCUP^2^. Among the last HF hospitalizations in patients’ timelines, 45,183 (16.6%) resulted in readmissions. Table 1 reports a general overview of the characteristics of the dataset including socio-demographics, hospitalization events, top diagnosis and procedures and the payment source. Table 2 reports the models’ performance in predicting the 30 days all-cause readmission for the last HF event in every patient’s timeline. Starting from RNN, the models trained with loss functions incorporating/emphasizing the loss from last HF event (i.e LastHF and Convex_HF_LastHF) achieved higher performance 0.636 and 0.635 AUC respectively compared to other loss function definitions. Moreover, RNN models with all four loss definitions achieved higher performance than RNNSS counterparts. For models incorporating CRF, the RNNCRF model achieved the highest performance with 0.642, followed by Neural CRF 0.634 and CRF only model achieving 0.63 with the first two models using pairwise potentials and the last one using unary potential. For convolutional models, CNN-Wide achieved better performance 0.632 compared to conventional CNN with 0.619. The MLP model achieved 0.628 placing it as the lowest performing model among the classes of neural models. The baseline model LR with *l*_1_-norm regularization (LASSO) achieved higher performance 0.643 compared to LR with *l*_2_-norm regularization 0.637. Models’ performance analysis is reported in Fig. 1 where panels A–E display the average ROC curve of the models across all 5-folds. Panel F in Fig. 1 compares the cumulative average AUC performance of the best models as a function of the patients’ timeline length. The analysis of feature importance is reported in Fig. 2, which shows the normalized coefficients of the trained LASSO models averaged across all folds. For the best neural model (RNNCRF), we report the analysis of feature importance using a similar approach to the one in^12^. In short, we iterated over all features attached to the last HF event, and computed the probability of readmission with a feature present or absent. Computing the difference between both probabilities allowed us to quantify a feature’s importance across the five folds. In the Supplementary Material section, we present additional variations on this technique. Overall, the average overlap (using Jaccard similarity) of the top-100 features between LASSO and the RNNCRF model is 51% and 55% for increase and decrease of readmission probability, respectively.

**Table 1:** Overview of HF dataset.

Variables	HF Dataset (n = 272,778)
**Socio-demographics**	
Age, mean (SD)	72.89 (14)
Gender female, count (%)	133765 (49%)
**Pay source, count (%)**	
Medicare	391535 (76.4%)
Private insurance	47327 (9.23%)
Medicaid	47095 (9.19%)
Self-pay	13115 (2.55%)
Other	11859 (2.31%)
No charge	1514 (0.29%)
**Hospitalization events**	
HF events, count (%)	343328 (66.94%)
days all-cause readmission, count (%)	81087 (23.61%)
Timeline length, mean (SD)	1.88 (1.4)
**Top 5 diagnosis, count (%)**	
Congestive heart failure; non-hypertensive	777047 (10.29%)
Coronary atherosclerosis and other heart disease	547890 (7.25%)
Residual codes	305406 (4.04%)
Cardiac dysrhythmias	298823 (3.95%)
Chronic kidney disease	254593 (3.37%)
**Top 5 procedures, count (%)**	
Diagnostic cardiac catheterization; coronary arteriography	106428 (14.95%)
Respiratory intubation and mechanical ventilation	57202 (8.03%)
Blood transfusion	52251 (7.34%)
Diagnostic ultrasound of heart (echocardiogram)	41076 (5.77%)
Hemodialysis	38083 (5.35%)

**Table 2:** Trained models’ performance based on the area under the ROC curve (AUC). CI: confidence interval.

Model name	AUC	CI - low	CI - high
CNN	0.619	0.616	0.622
CNN-Wide	0.632	0.629	0.635
RNN (Convex_HF_lastHF)	0.635	0.632	0.638
RNN (LastHF)	0.636	0.633	0.638
RNN (Uniform_HF)	0.631	0.628	0.634
RNN (Convex_HF_NonHF)	0.627	0.624	0.630
RNNSS (Convex_HF_lastHF)	0.621	0.618	0.624
RNNSS (LastHF)	0.625	0.623	0.628
RNNSS (Uniform_HF)	0.617	0.614	0.619
RNNSS (Convex_HF_NonHF)	0.625	0.622	0.628
Neural CRF (Pairwise)	0.634	0.631	0.637
Neural CRF (Unary)	0.631	0.629	0.634
CRF Only (Pairwise)	0.628	0.625	0.631
CRF Only (Unary)	0.630	0.627	0.633
RNNCRF (Pairwise)	**0.642**	0.640	0.645
RNNCRF (Unary)	0.638	0.635	0.641
MLP	0.628	0.625	0.631
Logistic regression *l*_2_-norm regularization	0.637	0.634	0.640
Logistic regression *l*_2_-norm regularization (LASSO)	**0.643**	0.640	0.646

## Discussion

This work highlights the advantages and limitations of deep learning in the domain of HF readmission prediction. As a first result, we observe that the inclusion of past hospitalization data improved prediction performance. In the medical field, this amounts to a unique opportunity to allow machines to base their predictions not only on the current status of a patient, but also on the patient’s history, and, if possible, on the comparative analysis to all patient data (present and historical) in a hospital system. Our data supports this notion, showing that the detailed past history, reflected in a timeline of patient hospitalization events, indeed carries additional information that boosts prediction performance. Not all models are born equal though, and for our study, we find that a scheduled sampling approach for RNN did not improve timeline-based predictions compared to vanilla RNN. Interestingly, pairing the RNN with a graphical model (CRF) resulted in the best performing neural model, an observation that mirrors previous results in the field of NLP^13,14^. Similarly, neural CRF performed better than CRF alone, hinting at the importance of adding nonlinear features to graphical models. While the actual performance numbers, with a maximal ROC score of 0.642 AUC (95% CI, 0.640–0.645), are inline with published machine-learning predictions of HF readmission^10,11^, it should be noted that they are based on administrative data, rather than rich EHR data as used in earlier studies. As such, our numbers represent the lower bound of achievable performance and deep learning on EHR data may eventually beat existing performance numbers for readmission prediction, as it did in other areas such as diagnosis and disease prediction^15,16^. Nevertheless, the exact approach for attaining better performance remains an open research question. One obvious avenue is to use multi-modal learning, incorporating several clinical data sources (including images), to offset the inherent issues with textual medical data, such as sparsity, missingness, and incompleteness. Our second result addresses the question of deep learning versus logistic regression for readmission prediction. Our face-off shows that logistic regression with regularization matches the best neural network performance. Our study attempted to compare these two approaches as fairly as possible, allowing both methods to perform hyperparameter optimization in the training phase, and giving logistic regression, which uses data from the last hospitalization event only, access to a patient’s hospitalization history by adding timeline summary data (such as number of HF events and number of admission events in the history) as an additional feature of the hospitalization event. Nevertheless, the LASSO model had a couple of advantages over the neural models by (1) having access to the whole training set during the hyperparameter optimization, and (2) using *l*_1_-norm regularization that served as feature-selection procedure while training the model. In contrast, the neural-based models had a very large set of hyperparameters to choose from (such as number of layers, dimensions of hidden vectors, etc.) that made it infeasible to explore the full hyperparameter space. We therefore opted for a random selection process for network configurations and optimization settings, and tested those against a subset of the training data only. Interestingly, and supporting our results, research by Rajkomar *et al*.^17^ on a more general hospital readmission problem (not focused on HF) also showed that logistic regression with regularization (LASSO) is competitive compared to an RNN model. Finally, our study sheds light on which features from the current or past hospitalizations are essential in readmission prediction. Features such as number of hospitalization events, length of stay, thrombophlebitis/thromboembolism and discharge against medical advice are among the highest contributing features to the log-odds of readmission (see Fig. 2). Generally, diagnoses and administered procedures pertaining to heart problems, such as contrast aortograms, result in increased readmission probability, as does the number of comorbidities. Interestingly, particular payment sources (Medicare and Medicaid) are associated with increased, while self-pay is associated with decreased readmissions.

In conclusion, using a large administrative data set, we show that neural network models and logistic regression (LASSO) have comparable performance on HF readmission prediction and that patient timeline data boosts prediction performance.

## Methods

### Dataset

The HF dataset was derived from the Healthcare Cost and Utilization Project (HCUP), Nationwide Readmission Database (NRD), issued by the Agency for Healthcare Research and Quality (AHRQ)^18^. It includes patients’ discharges (i.e. hospital claims) of all-payer hospital inpatient stays over the 2013 period that are contributed by twenty one states and accounting for 49.1% of all US hospitalizations^18^. Each claim in the dataset is associated with a corresponding patient who is identified by a uniquely generated linkage number (“*visitlink*”) that tracks the patient’s visits across hospitals within a state. Each claim represents a summary of an inpatients hospitalization event, including information about the hospitalization event such as the time of admission and discharge, the diagnosis, procedures, comorbidity and chronic conditions, length of stay, along other clinical fields associated with the event (a detailed description of the data elements can be found at^19^). Moreover, as each claim is linked to a patient identifier, it also includes patient’s socio-demographic information such as age, gender, income category and place location based on the National Center for Health Statistics (NCHS) classification scheme for US counties.

#### Timeline/trajectory building and processing

We built timelines/sequences out of the claims, allowing us to preserve the temporal progression and the history of hospitalization events for every patient. Patients were included in the HF dataset if they met the following conditions:

1. had at least one hospitalization event between January and November period with HF as the primary diagnosis (i.e. congestive heart failure; code = 108) as determined by Clinical Classification Software (CCS) that groups International Classification of Diseases, Version 9 (ICD-9) codes^20^
2. were ≥18 years old when they had an HF hospitalization event

Formally, we denote each claim (i.e. hospitalization event) by a feature vector ${\bar{x}}_{t}$ describing the characteristics and attributes of the hospitalization event and the corresponding patient. Moreover, we denote its corresponding label by *y*_*t*_ ∈ {0,1}, representing the 30 days all-cause readmission. The readmission outcome was computed based on the AHRQ HCUP 30-day readmission measure (see Appendix A in^21^).

To determine if *y*_*t*_ = 1 (i.e. the hospital admission of the future claim/event ${\bar{x}}_{t+1}$ occurs within 30 days from the current event ${\bar{x}}_{T}$), we traverse the patient’s timeline (temporally-ordered hospitalization events) from left to right and check if:

1. the current event ${\bar{x}}_{t}$ is an index event (i.e. an event where HF is the primary diagnosis as indicated by CCS diagnosis grouper; code = 108) and
2. the difference between the admission of the next event ${\bar{x}}_{t+1}$ and the discharge of current event ${\bar{x}}_{t}$ is ≤30 days (i.e. Δ*t* ≤ 30 days)

**Figure 3:**
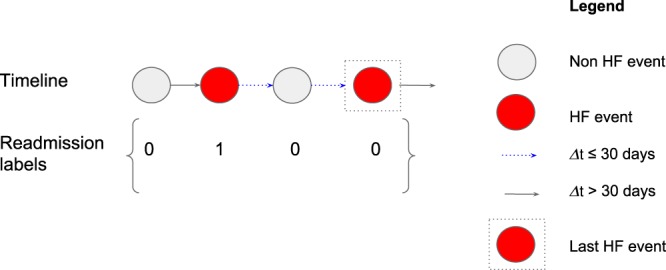
A toy example of a patient’s timeline with 30 days all-cause readmission labeling.

Figure 3 depicts the labeling process of a patient’s timeline. Notice the final event will always be the last HF event in a patient’s timeline for which we can determine its readmission label.

#### Dataset features

Each claim/event in a patient’s timeline was represented by a feature vector ${\bar{x}}_{t}$ encoding the characteristics of the hospitalization event and the corresponding patient. The feature vector included most of the fields included in the NRD databases describing every inpatients hospitalization event such as the time of admission and discharge, the diagnosis, procedures, comorbidity and chronic conditions, length of stay, along other clinical fields associated with the event. A detailed description of all the used features is found in the Supplementary Material.

### Models and notation

#### Sequence labeling and classification

In this section, we introduce the sequence labeling approach to 30 days all-cause readmission prediction. Generally, given a patient’s temporally ordered sequence of claims $\underline{{\bf{x}}}=[{\bar{x}}_{1},\ldots ,{\bar{x}}_{t},\ldots ,{\bar{x}}_{T}]$, represented by a *d*-dimensional feature vector ${\bar{x}}_{t}\in {{\mathbb{R}}}^{d}$, we seek a labeling $\underline{{\bf{y}}}$= [*y*_1_, …, *y*_*t*_, …, *y*_*T*_] representing the 30 days all-cause readmission outcomes where *y*_*t*_∈{0,1} and *T* is the patient-specific sequence length (i.e. equivalent to *T*_*i*_ where *i* refers to the *i*-th patient in training dataset). Given a training set ${D}_{train}={\{(\underline{{{\bf{x}}}_{i}},\underline{{{\bf{y}}}_{i}})\}}_{i=1}^{N}$, the goal is to learn a model (i.e. function map *f*) by minimizing an objective function *L*(*f*, *D*_*train*_) that measures the discrepancy between every sequence’s target labels ${\underline{{\bf{y}}}}_{i}$ and its corresponding predicted label sequence ${\underline{\hat{{\bf{y}}}}}_{i}$ in the training dataset. A common approach is to use a parametrized function *f*(***θ***) such that learning the best function map (i.e. training a model) translates into finding the optimal weights ***θ*** where ***θ*** = argmin_***θ***_*L*(*f*, *D*_*train*_). With the choice of a differentiable function, the optimal weights ***θ*** are obtained through an iterative process by using the gradient of the objective function ▽_***θ***_*L*(*f*, *D*_*train*_), scaling it with step size *η*, and subtracting the result from the current weights at each iteration. Intuitively, the weights update equation

1 $${{\boldsymbol{\theta }}}^{k+1}={{\boldsymbol{\theta }}}^{k}-\eta {\nabla }_{{{\boldsymbol{\theta }}}^{k}}L(f,{D}_{train})$$

is directing the new weights toward the steepest descent (i.e. the direction which minimizes *L*(*f*, *D*_*train*_)) at each update iteration *k*. Sequence classification is similar to sequence labeling but instead of assigning labels/classes to each event in the sequence, we assign one single label/class to the whole sequence. Thus, in the sequence labeling setting, the trained model will predict an outcome for every event in the sequence, while in the sequence classification setting, the model predicts the class of the whole sequence. In this work, the difference between both approaches is mainly in the training phase (learning the labels of all events versus one single label for the sequence), while during the testing phase, both models are used to predict the outcome/label of the last HF event. The latter is directly provided through sequence labeling. In sequence classification, we are using the label of the last HF event as a substitute for the sequence label and a training loss that is associated with that event. To summarize, we use the term “labeling” and “classification” to differentiate between models incorporating the labels of previous events in the training/learning of the model versus optimizing only on the last HF event label. In all cases, the testing/decoding phase is equivalent and is focused on the prediction of the label/class of the last HF event.

#### Objective function

We defined the loss at each time step for an *i*-th sequence by the cross-entropy loss

2 $${l}_{t}^{(i)}=-\sum _{c=1}^{|{V}_{label}|}\,{y}_{t,c}^{(i)}\times log({\hat{y}}_{t,c}^{(i)})$$

where *V*_*label*_ is set of admissible classes, |*V*_*label*_| is the number of classes, *y*_*t*,*c*_ ∈ {0,1} is equivalent to 𝟙[*y*_*t*_ = *c*] (i.e. a boolean indicator that is equal to 1 when *c* is the reference/ground-truth class at time *t*), and ${\hat{y}}_{t,c}$ is the probability of the class *c* at time *t*. Four realizations/definitions of objective functions were tested in this study. Given that our focus is on the 30 days all-cause readmissions for the last HF hospitalization event, the first loss (Convex_HF_lastHF) was defined by a convex combination between the average loss from all HF events in patient’s timeline and the loss from the last HF event. The convex combination is parametrized by parameter *α* that was determined using a validation set inspired by the work done in^16^. The second loss function (LastHF) used the loss computed only from the last HF event while the third (Uniform_HF) uniformly averaged the loss from all HF events in a patient’s timeline. Lastly, the forth objective function (Convex_HF_NonHF) was based on a convex combination between the average loss contributed by all HF events in patient’s timeline and the average loss from the non HF events.

The objective function for the whole training set *D*_*train*_ was defined by the average loss *L*_*i*_ across all the sequences in *D*_*train*_ plus a weight regularization term *λ* applied to the model parameters represented by ***θ*** (i.e. *l*_2_-norm regularization)

3 $${L}_{i}=\frac{1}{{T}_{i}}\sum _{t=1}^{{T}_{i}}\,{l}_{t}^{(i)}$$

4 $$L({\boldsymbol{\theta }})=\frac{1}{N}\sum _{i=1}^{N}\,{L}_{i}+\frac{\lambda }{2}||{\boldsymbol{\theta }}|{|}_{2}^{2}$$

In addition to the *l*_2_-norm regularization, we also experimented with dropout^22^ by deactivating neurons in the network layers using probability *p*_*dropoout*_.

#### Recurrent neural network (RNN)

Recurrent neural networks (RNN) is a connectionist model that is well suited for modeling sequential and temporal data with varying length^23–25^. A basic RNN is similar to feed-forward neural network but with additional support for cyclical connections (i.e. recurrent edges among the hidden layers at different time steps)^24,25^. RNN computes a hidden vector at each time step (i.e. state vector ${\bar{h}}_{t}$ at time *t*), representing a history or context summary of the sequence using the input and hidden states vector form the previous time step. This allows the model to learn long-range dependencies where the network is unfolded as many times as the length of the sequence it is modeling. To compute the outcome ${\hat{y}}_{t}$, an affine transformation followed by non-linear activation function *σ* is applied to the state vector ${\bar{h}}_{t}$. The non-linear operator *σ* can be either the *sigmoid* function applied to a scalar input or its generalization the *softmax* function applied to vector. As a result, the outcome ${\hat{y}}_{t}$ represents a probability distribution over the set of possible labels at time *t*. Gradient descent (i.e. “*vanilla*” gradient descent as in Eq. 1 or any variant) is used for optimizing the weights of the network while the gradient is computed using back propagation through time^26^. Although RNNs are capable of handling and representing variable-length sequences, in practice, the learning process faces challenges due to the vanishing/exploding gradient problem^24,27,28^. To overcome these challenges, gradient clipping^29^ and gated memory cells approach as in long short-term memory (LSTM) and gated recurrent unit (GRU)^30–32^ were proposed replacing the conventional nodes in the hidden layer and hence updating the computation mechanism of the hidden state vector ${\bar{h}}_{t}$.

#### RNN with scheduled sampling (RNNSS)

Another variation of the RNN model that we experimented with is using a scheduled sampling approach (“teacher forcing”)^33^ while training the RNN model. The approach trains a model using predicted labels from the model itself, in addition to the (true) reference labels. The process starts by training based on the true labels and, as the training progresses, progressively based on the predicted labels^33^. The choice between using true labels versus predicted labels is controlled by a probability parameter *ε*. The authors in^33^ proposed a scheduling where *ε* is initially set to 1 (i.e. the model uses only the true labels) with subsequent decrease in *ε* as the training progresses. In this work, we explored the use of linear, exponential, and inverse sigmoid scheduling decay. Moreover, we trained the RNN-based models using the four definitions of the loss function detailed in the ***Objective function*** section.

#### Conditional random fields (CRF)

Although RNN models are suited for modeling temporal data, the outcome/label prediction for each event is preformed independently from each other. That is: the labeling decision is done *locally* (i.e. without considering any association/correlation between neighboring labels). In other words, there is a need for a joint modeling approach that is *global* by considering the whole sequence of labels when performing the optimization and inference. Linear-chain CRF suits this requirement well by modeling the probability of the whole labeled sequence (i.e. outcome sequence) given the input sequence. It is a class of undirected discriminative graphical models that uses a global feature function within a log-linear model formalism, making it well suited for structured prediction^34,35^. In this study, we applied CRF in two occasions with two variations (i.e. definition of potential functions).

#### CRF with RNN

We first experimented with combining the RNN model with a CRF layer by feeding the computed features from the RNN layer as inputs to the CRF layer as in^13^. We denote the output features of the RNN layer by $\underline{{\bf{z}}}=[{\bar{z}}_{1},{\bar{z}}_{2},\cdots ,{\bar{z}}_{T}]$ representing the sequence of output features computed from the input sequence $\underline{{\bf{x}}}$ (both sequences have equal length). The potential functions in the CRF layer were computed using $\underline{{\bf{z}}}$ along with label sequence $\underline{{\bf{y}}}$ in two variations:

1. RNNCRF (Unary) that computed unary potential by using only the RNN output feature vector to generate an output vector with dimension equal to the number of classes |*V*_*label*_| for each ${\bar{z}}_{t}$. The pairwise potential is modeled using a transition parameters matrix *A*(*y*_*t*−1_, *y*_*t*_) of size |*V*_*label*_| × |*V*_*label*_| representing the transition score from one outcome class to another.
2. RNNCRF (Pairwise) that computes pairwise potentials using both the RNN output feature vectors and the labels sequence such that it generates an output vector of size |*V*_*label*_| × |*V*_*label*_| at every time step *t* similar to the approach reported in^14^.

We provide further details regarding the CRF model formulation and potential function computation in the Supplementary Material section.

#### CRF & Neural CRF

We also tested a CRF approach without the RNN block. The first used CRF only (i.e. first-order linear chain CRF) model using the two variations of potential functions (i.e. unary and pairwise). The second model is combining CRF with neural model (i.e. using non-linear transformation for computing features) similar to the approach in^36^ using the same two potential function variants. The objective function for models that incorporated CRF was defined by the negative conditional log-likelihood *L*(***θ***) plus an *l*_2_-norm weight regularization term,

5 $$L({\boldsymbol{\theta }})=[\frac{1}{N}\sum _{i=1}^{N}\,-\,log(p(\underline{{{\bf{y}}}_{{\bf{i}}}}|\underline{{{\bf{x}}}_{{\bf{i}}}}))]+\frac{\lambda }{2}||{\boldsymbol{\theta }}|{|}_{2}^{2}$$

Estimating the optimal weights ***θ*** is typically done by applying a variant of gradient descent algorithm (as described in Eq. 1) where the sum-product algorithm (i.e. performing a variation of the forward-backward algorithm^37^) is used. Decoding the sequence (i.e. finding the optimal labeling ${\underline{{\bf{y}}}}_{optimal}$) is done through a variant of Viterbi algorithm^38,39^.

#### Convolutional neural networks (CNN)

The CNN models adopt the sequence classification view by using the 2 *D* arrangement of the patients’ timelines with an objective function defined only for the last HF event (i.e. the loss function is defined for the last HF event that we seek to predict its readmission outcome). A CNN model is a feed-forward neural network that typically consists of multiple layers of which *convolutional* layer is the building block. A convolutional layer is composed of filters/kernels (in our context, the kernel is a 2 *D* arrangement of weights in matrix form) that are convolved with the features of the previous layer (such as the input layer) to produce feature maps. More formally, a patient’s timeline was arranged in a matrix form where the sequence of events are stacked (i.e. concatenated) to form a matrix $X={[{\bar{x}}_{1}{\bar{x}}_{2}\cdots {\bar{x}}_{{T}_{max}}]}^{\top }$ of size *T*_*max*_ × *d* where *d* is the dimension of an event vector ${\bar{x}}_{t}$ and *T*_*max*_ is the maximum length of a patient’s timeline in the training set – patients with shorter timelines are padded to have a common representation. A kernel *F* is a matrix of weights that is convolved with *X* to produce a feature map *M* such that an entry in *M* is computed by first taking the sum of element-wise multiplication of the weights in the kernel *F* and the corresponding input of the previous layer, then adding a bias term followed by non-linear operation. Typically, multiple kernels are applied and the resulting feature maps are stacked on top of each other forming a 3 *D* volume/tensor to be processed subsequently in the next layers. The weights in each kernel represent the shared parameters that we optimize during the training phase. Another type of layers in this network is a *pooling* layer that also includes kernels/filters but with no trainable weights, which slides over the input feature maps based on a defined horizontal and vertical stride size and computes a summary score such as a maximum or average score for every region of overlap. As a result, in the pooling layer we can change the size of the generated feature maps by specifying the stride and padding size such that the size of the feature maps decreases as we progress into subsequent layers in the network (i.e. equivalent to subsampling). Another commonly used layer after the convolutional/pooling layers is the *fully-connected* layer (FC). FC takes an input vector from the reshaped feature maps generated in the last convolutional/pooling layers and applies an affine transformation followed by non-linear element-wise operation. In this work, we experimented with two types of convolutional models:

1. CNN model that describes a network inspired by commonly used models in computer vision and image processing research^40^ that makes use of multiple square convolutional and pooling kernels (i.e. 2 × 2, 3 × 3, 5 × 5), where the generated feature maps are reduced in size as a function of the network depth (i.e. number of layers) until reaching to the fully-connected layer/s.
2. CNN-Wide model that adapts the approach used by Kim^41^ for sentence classification where the convolutional kernels are wide/rectangular covering the whole input feature dimension. In other words, a kernel in this model would have varying sizes (such as 2 × *d*, 3 × *d*, 5 × *d*) where the convolution is applied to the whole feature vector for two or more events for every possible window of events in the patient’s timeline (i.e. applied to matrix *X*). After each convolution operation, the result is a vector of feature map corresponding to one kernel. In this network, the pooling layer reduces each generated feature map vector to a scalar (i.e. one feature) and then concatenates each one of them into one vector having number of elements equal to the number of applied convolutional kernels. Lastly, the resulting vector is passed into one or more FC layers before it is passed to the output layer.

Both CNN models use an output layer where the computed vector of activations/feature map in the penultimate layer are passed to generate a probability distribution over the outcome labels (as in the RNN case). The defined loss function in both models is the loss computed for the last HF event (see LastHF in the ***Objective function*** section). The overall objective function for the training set is defined in Eq. 4.

#### Multilayer perceptron (MLP)

A final neural network-based model is the multilayer perceptrons which is also a feed-forward neural network (MLP). The MLP network is composed of an input layer then a set of multiple FC layers and lastly an output layer that generates a probability distribution over the outcome classes. The FC layers, as we discussed earlier, mainly consists of two operations; an affine transformation followed by non-linear element-wise operation to generate new feature vectors (i.e. learned representations). The difference between this modeling approach and the previous ones is that MLP takes the *event* view of the problem by modeling the last index event ${\bar{x}}_{T}$ only and discarding the sequence aspect of the patients’ timeline. The defined loss is computed based on the last HF event (i.e. LastHF definition) and the overall objective function is defined in Eq. 4.

#### Logistic regression (LR)

Logistic regression (LR) is a commonly used model for classification problems due to its simplicity and model interpretability. Like MLP, LR supports the *event* view of the problem by modeling only the last index event. LR model can be considered as a neural network model with no hidden layers and one output neuron. In this setup, the input features are fully-connected to one output neuron where the *sigmoid* function is applied as a non-linear operation computing the probability of the outcome label to be equal to 1. In other words, the LR model computes $p({\hat{y}}_{T}=1|{\bar{x}}_{T})=\frac{1}{1+{\exp }^{-({{\bf{W}}}_{1\times {\bf{d}}}{\bar{{\bf{x}}}}_{{\bf{T}}}+{\bf{b}})}}$ where *W*_1×*d*_ is the weight matrix that maps ${\bar{x}}_{T}$ to a scalar value (i.e. using one neuron), *b* is the bias term and $\frac{1}{1+{\exp }^{-z}}$ is the *sigmoid* function representing the non-linear operation. The output represents the probability of a patient readmitting to hospital within 30 days after HF hospitalization event. In this work, LR was the baseline model that we compare its performance to the ones of the neural network-based models. The loss function for each patient’s last event is defined by the conditional log-likelihood which is equivalent to the cross-entropy loss for the binary case (i.e 2-class classification) and the overall objective function is based on the average conditional log-likelihood of the data (see Eq. 4). Additionally, we experimented with two regularization schemes: (1) *l*_1_-norm regularization (LASSO) and (2) *l*_2_-norm regularization.

## Experimental Setup

We followed a stratified 5-fold cross-validation scheme, in which the HF dataset is split into 5 folds, each having a training and test set size of 80% and 20% of the data, respectively, and a validation set size of 10% of the training set in each fold (used for optimal epoch selection in case of neural models or hyperparameter selection in case of logistic regression). Moreover, due to the imbalance in outcome classes (i.e. no readmission vs. readmission), training examples were weighted inversely proportional to class/outcome frequencies in the training data. The models’ performance was evaluated using the last HF event in the patients’ timeline (i.e. 30 days all-cause readmission after hospitalization for HF event). We used the area under the ROC curve (AUC) as our performance measure with confidence intervals computed using the approach reported in LeDell *et al*.^42^. The optimal cutoff  was computed for each model for every fold using the Youden-Index and then averaged across all 5-folds to determine the optimal cutoff position with the ± standard deviation. Moreover, the evaluation of the trained models was based on their average performance on the test sets of the five folds.

### Hyperparameter optimization for neural models

Neural model hyperparameter selection is costly, particularly for finding the optimal architecture. To this end, we randomly chose one fold where 30% of the training set was further split into a training and validation set, each having 90% and 10% of the data, respectively. We developed a multiprocessing module that used a uniform random search strategy^43^ that randomly chose a set of hyperparameters configurations (i.e. layer depth, filter size and optimization methods, see Supplementary Materials for more details) from the set of all possible configurations. Then the best configuration for each model (i.e. the one achieving best performance on the validation set) was used for the final training and testing.

## Supplementary information

Supplementary material

## Data Availability

The dataset was derived from the Healthcare Cost and Utilization Project (HCUP), Nationwide Readmission Database (NRD), issued by the Agency for Healthcare Research and Quality (AHRQ) for use as limited dataset. Recipients of the dataset are required to go through a HCUP Data Use Agreement (DUA) course and sign the DUA before receiving the data. The data storing, handling, analysis and reporting for this study was done in accordance with the DUA. Moreover, the code for data processing, model implementation, training and testing workflow is  publicly available at the following link: https://bitbucket.org/A_2/hcup_research.
