# Supplementary material for: Neural networks versus Logistic regression for 30 days all-cause readmission prediction
Source: Sci Rep. 2019 Jun 26;9:9277. doi: 10.1038/s41598-019-45685-z (PMC6595068; doi:10.1038/s41598-019-45685-z)
Supplement: Supplementary file 1 — Supplementary material [file 41598_2019_45685_MOESM1_ESM.pdf]

Supplementary Material for Neural networks versus Logistic regression for 30 days all-cause readmission prediction

Ahmed Allam*[1,2], Mate Nagy†[3], George Thoma‡[5], and Michael Krauthammer§[1,2,3,4]

[1]Department of Quantitative Biomedicine, University of Zurich
[2]Chair of Medical Informatics, University Hospital of Zurich
[3]Program in Computational Biology and Bioinformatics, Yale University School of Medicine
[4]Department of Pathology, Yale University School of Medicine
[5]Lister Hill National Center for Biomedical Communications, National Library of Medicine

*ahmed.allam@uzh.ch
†mate.nagy@yale.edu
‡gthoma@mail.nih.gov
§michael.krauthammer@uzh.ch

1 Methods

1.1 Recurrent neural network (RNN)

RNN computes a hidden vector at each time step (i.e. state vector $\overline{h}_t$ at time t), representing a history or context summary of the sequence using the input and hidden states vector form the previous time step. This allows the model to learn long-range dependencies where the network is unfolded as many times as the length of the sequence it is modeling. Equation 1 shows the computation of the hidden vector $\overline{h}_t$ using the input $\overline{x}_t$ and the previous hidden vector $\overline{h}_{t-1}$ where ϕ is a non-linear transformation such as $ReLU(z) = max(0, z)$ or $tanh(z) = \frac{e^z - e^{-z}}{e^z + e^{-z}}$. To compute the outcome $\hat{y}_t$ at time t, an affine transformation followed by non-linear function are applied to the state vector $\overline{h}_t$ as described in equation 2. The non-linear operator σ can be either the *sigmoid* function $\sigma(z) = \frac{1}{1+e^{-z}}$ applied to scalar input $z \in \mathbb{R}$, or its generalization the *softmax* function applied to vector $\overline{z} \in \mathbb{R}^K$, $softmax(\overline{z})_i = \frac{e^{z_i}}{\sum_{j=1}^{K} e^{z_j}}$ for $i = 1, ..., K$. As a result, the outcome $\hat{y}_t$ represents a probability distribution over the set of possible labels V_{label} at time t.

$$\overline{h}_t = \phi(\mathbf{W}_{hx}\overline{x}_t + \mathbf{W}_{hh}\overline{h}_{t-1} + \overline{b}_{hx}) \tag{1}$$

$$\hat{y}_t = \sigma(\mathbf{W}_{V_{label}h}\overline{h}_t + \overline{b}_{V_{label}}) \tag{2}$$

where $\mathbf{W}_{hh} \in \mathbb{R}^{D_h \times D_h}$, $\mathbf{W}_{hx} \in \mathbb{R}^{D_h \times d}$, $\mathbf{W}_{V_{label}h} \in \mathbb{R}^{|V_{label}| \times D_h}$, $\overline{b}_{hx} \in \mathbb{R}^{D_h}$, $\overline{b}_{V_{label}} \in \mathbb{R}^{|V_{label}|}$ representing the model weights $\boldsymbol{\theta}$ to be optimized and D_h, d are the dimensions of $\overline{h}_t$ and $\overline{x}_t$ vectors respectively. Note that the weights are shared across all the network (see Figure 1 for RNN representation).

1.1.1 Long short-term memory (LSTM)

Long short-term memory (LSTM) [1,2] falls in the gated memory cells approach that modifies the basic RNN by replacing the standard neurons/units in the hidden layer with gated/memory cells to generate the hidden state vector $\overline{h}_t$ as described by the equations below. Moreover, LSTM introduces a new cell state vector $\overline{c}_t$ that overall contributes in the decision mechanism on what part of the history to keep or forget. The computation of the output $\hat{y}_t$ at time t remains the same as explained in the RNN section.

$$\overline{i}_t = \sigma(\mathbf{W}^i_{hx}\overline{x}_t + \mathbf{W}^i_{hh}\overline{h}_{t-1} + \overline{b}^i_{hx}) \quad \text{(input gate)}$$
$$\overline{f}_t = \sigma(\mathbf{W}^f_{hx}\overline{x}_t + \mathbf{W}^f_{hh}\overline{h}_{t-1} + \overline{b}^f_{hx}) \quad \text{(forget gate)}$$
$$\overline{o}_t = \sigma(\mathbf{W}^o_{hx}\overline{x}_t + \mathbf{W}^o_{hh}\overline{h}_{t-1} + \overline{b}^o_{hx}) \quad \text{(output gate)}$$
$$\overline{\tilde{c}}_t = \phi(\mathbf{W}^{\tilde{c}}_{hx}\overline{x}_t + \mathbf{W}^{\tilde{c}}_{hh}\overline{h}_{t-1} + \overline{b}^{\tilde{c}}_{hx}) \quad \text{(new state/memory cell)}$$
$$\overline{c}_t = \overline{f}_t \odot \overline{c}_{t-1} + \overline{i}_t \odot \overline{\tilde{c}}_t \quad \text{(final cell state)}$$
$$\overline{h}_t = \overline{o}_t \odot \phi(\overline{c}_t) \quad \text{(hidden state vector)}$$

where $\mathbf{W}^i_{hx}$, $\mathbf{W}^f_{hx}$, $\mathbf{W}^o_{hx}$, $\mathbf{W}^{\tilde{c}}_{hx}$ each $\in \mathbb{R}^{D_h \times d}$ and $\mathbf{W}^i_{hh}$, $\mathbf{W}^f_{hh}$, $\mathbf{W}^o_{hh}$, $\mathbf{W}^{\tilde{c}}_{hh}$ each

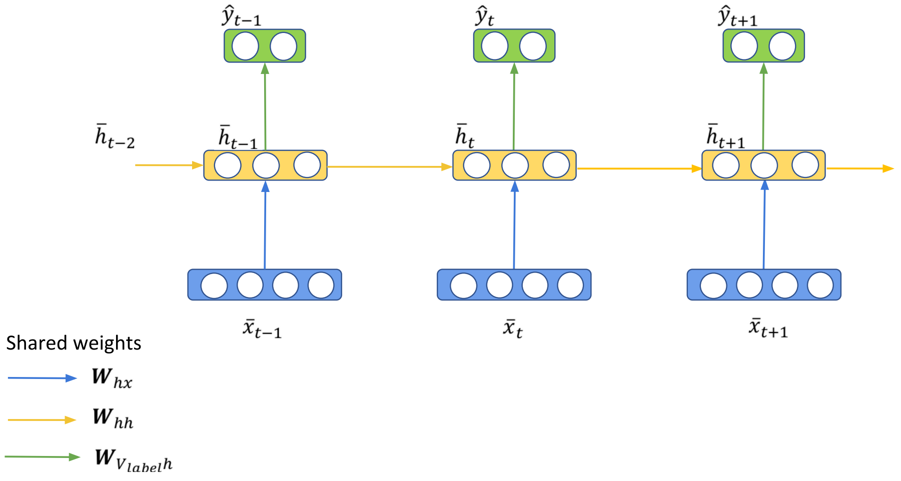

Figure 1: Graphical representation of unfolded RNN

$\in \mathbb{R}^{D_h \times D_h}$. The biases $\bar{b}^i_{hx}$, $\bar{b}^f_{hx}$, $\bar{b}^o_{hx}$, $\bar{b}^{\tilde{c}}_{hx}$ each $\in \mathbb{R}^{D_h}$ where D_h and d are the dimensions of $\bar{h}_t$ and $\bar{x}_t$ vectors respectively. The operator σ represents the *sigmoid* function, ϕ the *tanh* or *ReLU* function, and $\odot$ the element-wise product (i.e. Hadamard product) function. Compared to the standard/conventional RNN (see Equation 1), it can be noted the added complexity in terms of the number of weight matrices and biases required to compute the hidden state vector $\bar{h}_t$.

1.1.2 Gated recurrent unit (GRU)

Gated recurrent unit (GRU) [3] presents similar approach to LSTM but with a simpler model that modifies the computation mechanism of the hidden state vector $\bar{h}_t$ through the specified equations below.

$$\bar{z}_t = \sigma(\mathbf{W}^z_{hx}\bar{x}_t + \mathbf{W}^z_{hh}\bar{h}_{t-1} + \bar{b}^z_{hx}) \qquad \text{(update gate)}$$
$$\bar{r}_t = \sigma(\mathbf{W}^r_{hx}\bar{x}_t + \mathbf{W}^r_{hh}\bar{h}_{t-1} + \bar{b}^r_{hx}) \qquad \text{(reset gate)}$$
$$\bar{\tilde{h}}_t = \phi(\mathbf{W}^{\tilde{h}}_{hx}\bar{x}_t + \bar{r}_t \odot \mathbf{W}^{\tilde{h}}_{hh}\bar{h}_{t-1} + \bar{b}^{\tilde{h}}_{hx}) \qquad \text{(new state/memory cell)}$$
$$\bar{h}_t = (1 - \bar{z}_t) \odot \bar{\tilde{h}}_t + z_t \odot h_{t-1} \qquad \text{(hidden state vector)}$$

The model computes a reset gate $\bar{r}_t$ that is used to modulate the effect of the previous hidden state vector $\bar{h}_{t-1}$ when computing the new memory vector $\bar{\tilde{h}}_t$. The update gate $\bar{z}_t$ determines the importance/contribution of the newly generated memory vector $\bar{\tilde{h}}_t$ compared to the previous hidden state vector $\bar{h}_{t-1}$ when

computing the current hidden vector $\overline{h}_t$. The weights $\mathbf{W}^{z}_{hx}$, $\mathbf{W}^{r}_{hx}$, $\mathbf{W}^{\tilde{h}}_{hx}$ each $\in \mathbb{R}^{D_h \times d}$ and $\mathbf{W}^{z}_{hh}$, $\mathbf{W}^{r}_{hh}$, $\mathbf{W}^{\tilde{h}}_{hh}$ each $\in \mathbb{R}^{D_h \times D_h}$. The biases $\overline{b}^{z}_{hx}$, $\overline{b}^{r}_{hx}$, $\overline{b}^{\tilde{h}}_{hx}$ each $\in \mathbb{R}^{D_h}$ where D_h and d are the dimensions of $\overline{h}_t$ and $\overline{x}_t$ vectors respectively. The operators notation have the same meaning as described in the LSTM section.

1.1.3 RNN objective function

In our work, we use RNN to refer to RNN, LSTM and GRU models targeting the sequence labeling view of the problem. We defined the loss for an i-th sequence at each time step by the cross-entropy loss

$$l_t^{(i)} = -\sum_{c=1}^{|V_{label}|} y_{t,c}^{(i)} \times log(\hat{y}_{t,c}^{(i)}) \tag{3}$$

where the loss for the i-th sequence (i.e. i-th patient's timeline/trajectory) is defined by the average loss over the sequence length T_i

$$L_i = \frac{1}{T_i} \sum_{t=1}^{T_i} l_t^{(i)} \tag{4}$$

Given that our focus is on the 30 days all-cause readmissions after HF hospitalization, our model's focus should be on the index events (i.e. claims/events where HF is the primary diagnosis of hospitalization). Hence, the objective function could be modified to reflect this requirement. We modify the defined loss over i-th sequence (see Eq. 4) by defining an average loss for non-index and index events separately and then taking a convex combination between both losses parametrized by α. The parameter α is determined using a validation set. Our modification is inspired by the work done in [4].

$$L_i^{HF} = \frac{1}{\sum_{t=1}^{T_i} \mathbb{1}[\overline{x}_{t,primaryHF}^{(i)} = 1]} \sum_{t=1}^{T_i} l_t^{(i)} \mathbb{1}[\overline{x}_{t,primaryHF}^{(i)} = 1]$$

$$L_i^{nonHF} = \frac{1}{\sum_{t=1}^{T_i} \mathbb{1}[\overline{x}_{t,primaryHF}^{(i)} = 0]} \sum_{t=1}^{T_i} l_t^{(i)} \mathbb{1}[\overline{x}_{t,primaryHF}^{(i)} = 0]$$

$$L_i = (1-\alpha) L_i^{nonHF} + \alpha(L_i^{HF}) \tag{5}$$

where $\mathbb{1}[\overline{x}_{t,primaryHF}^{(i)} = 1]$ is an indicator function that is equal to 1 when the feature vector $\overline{x}_t^{(i)}$ representing the event at time t for the i-th sequence has HF as the primary diagnosis (we refer to this loss by Convex_HF_NonHF). A second variation (Uniform_HF) to the first objective function in Eq. 5, is to consider only the index events in the patients' timeline where we compute the average cross-entropy loss for the index events only.

$$L_i = L_i^{HF} \tag{6}$$

A third variation (Convex_HF_lastHF) is to take a convex combination between all index events and the last index event in the timeline.

$$L_i^{HF} = \frac{1}{\sum_{t=1}^{T_i} \mathbb{1}[\overline{x}_{t,primaryHF}^{(i)} = 1]} \sum_{t=1}^{T_i} l_t^{(i)} \mathbb{1}[\overline{x}_{t,primaryHF}^{(i)} = 1]$$

$$L_i^{lastHF} = l_T^{(i)}$$

$$L_i = (1-\alpha) L_i^{HF} + \alpha (L_i^{lastHF}) \tag{7}$$

where L_i^{lastHF} is the cross-entropy loss for the last HF event (i.e. the last index event in the patient's timeline) and $l_T^{(i)} = -\sum_{c=1}^{|V_{label}|} y_{T,c}^{(i)} \times log(\hat{y}_{T,c}^{(i)})$.
The previous variations consider the *sequence labeling* approach since the loss function for a sequence is defined as a composite of losses from different times/events in a patient's timeline. A final variation that uses the *sequence classification* view of the problem is to define an objective function that focuses only on the last HF event (LastHF) by computing the loss at the last target event we aim to predict.

$$L_i = L_i^{lastHF} \tag{8}$$

Lastly, the objective function for the whole training set D_{train} is defined by the average loss across all the sequences in D_{train} plus a weight regularization term (i.e. l_2-norm regularization) applied to the model parameters represented by $\boldsymbol{\theta}$

$$L(\boldsymbol{\theta}) = \frac{1}{N} \sum_{i=1}^{N} L_i + \frac{\lambda}{2} ||\boldsymbol{\theta}||_2^2 \tag{9}$$

In practice, the training occurs using mini-batches where computing the loss function and updating the parameters/weight occur after processing each mini-batch of the training set.

1.1.4 RNN with conditional random fields (CRF)

CRF models the conditional probability of a sequence $\underline{\mathbf{y}}$ given its corresponding sequence of observation vectors $\underline{\mathbf{x}}$ (i.e. $p(y_1, y_2, ..., y_T | \overline{x}_1, \overline{x}_2, ..., \overline{x}_T)$) using a parametrized *global* feature vector $\overline{F}(\underline{\mathbf{x}},\underline{\mathbf{y}}) \in \mathbb{R}^J$ that takes input/output sequences to produce J-dimensional vector. As a result, the computation of the conditional probability of an output sequence given its input sequence of observations is equal to

$$p(\underline{\mathbf{y}}|\underline{\mathbf{x}}) = \frac{e^{\boldsymbol{\theta} \cdot \overline{F}(\underline{\mathbf{x}},\underline{\mathbf{y}})}}{\sum_{\underline{\mathbf{y}}' \in \underline{\mathbf{Y}}} e^{\boldsymbol{\theta} \cdot \overline{F}(\underline{\mathbf{x}},\underline{\mathbf{y}}')}} \tag{10}$$

where $\underline{\mathbf{Y}}$ is the set of all label sequences, the denominator represents the normalizer (commonly referred to the partition function Z), and $\boldsymbol{\theta}$ is the weight vector corresponding to the global feature vector $\overline{F}(\underline{\mathbf{x}},\underline{\mathbf{y}})$. The common definition of the feature vector $\overline{F}$ uses the first-order Markov assumption in order to make the inference and model training tractable [5]. That is

$$\overline{F}(\underline{\mathbf{x}},\underline{\mathbf{y}}) = \sum_{t=1}^{T} \overline{f}(\underline{\mathbf{x}}, t, y_{t-1}, y_t) \quad (11)$$

where the global feature vector $\overline{F}$ is the sum of a local feature vector $\overline{f}$ applied at each time step until the end of the sequence. The local vector $\overline{f}$ has the same dimension of $\overline{F}$ (i.e. $\in \mathbb{R}^J$) and has access to the whole observation sequence $\underline{\mathbf{x}}$, and the current and previous states/outputs y_{t-1} and y_t [5]. Generally, increasing the model order k (i.e. $k \geq 2$) would lead to exponential computational complexity in terms of k. However, recent work as in [6–8], showed under the assumption of label pattern sparsity, the use of higher-order models (i.e. models with $k \geq 2$) is feasible without incurring an exponential complexity in the training and inference algorithms [9].
We denote the output features of the RNN layer by $\underline{\mathbf{z}} = [\overline{z}_1, \overline{z}_2, \cdots, \overline{z}_T]$ representing the sequence of output features computed from the input sequence $\underline{\mathbf{x}}$ (both sequences have equal length). The potential functions in the CRF layer are computed using $\underline{\mathbf{z}}$ along with label sequence $\underline{\mathbf{y}}$. In our work, we experimented with two potential functions:

1. RNNCRF (Unary) that computes unary potentials $\psi_{y_t}(\overline{z}_t)$ by passing the RNN output feature vector $\overline{z}_t$ at time t to a linear affine map and applying a non-linear transformation resulting in a vector of size equal to the number of classes $|V_{label}|$ for each $\overline{z}_t$. The pairwise potential is modeled using a transition parameters matrix $A(y_{t-1}, y_t)$ of size $|V_{label}| \times |V_{label}|$ representing the transition score from one outcome class to another. The total score computation is equal to $\overline{F}(\underline{\mathbf{z}},\underline{\mathbf{y}}) = \sum_{t=1}^{T}(\psi_{y_t}(\overline{z}_t) + A(y_{t-1}, y_t))$.

2. RNNCRF (Pairwise) that computes pairwise potentials $\psi_{y_{t-1}y_t}(\overline{z}_t)$ by using linear affine map transformation followed by non-linear element-wise operation generating an output of size $|V_{label}| \times |V_{label}|$ similar to the approach reported in [10]. The total score $\overline{F}(\underline{\mathbf{z}}, \underline{\mathbf{y}})$ is equal to $\overline{F}(\underline{\mathbf{z}},\underline{\mathbf{y}}) = \sum_{t=1}^{T} \psi_{y_{t-1}y_t}(\overline{z}_t)$.

1.2 Dataset features representation

1.2.1 Input features $\overline{x}_t$

Each claim/event in a patient's timeline is represented by a feature vector $\overline{x}_t$ encoding the characteristics of the hospitalization event and the corresponding patient. The feature vector $\overline{x}_t$ is composed of:

- Diagnosis: every claim in the dataset includes 25 ordered fields, each registering patient's diagnosis category based on CCS grouper [11] during the corresponding hospitalization event. We first extracted set $V_{diagnosis}$ representing the diagnosis having at least 1000 counts/occurrences registered in the HF dataset. Then, we constructed the following vectors:

    1. $\overline{x}_{diag1}$ a one-hot encoded vector $\in \{0,1\}^{|V_{diagnosis}|}$ representing the diagnosis category registered for the primary diagnosis field
    2. $\overline{x}_{diag2}$ a one-hot encoded vector $\in \{0,1\}^{|V_{diagnosis}|}$ representing the diagnosis category registered for the secondary diagnosis field

3. $\overline{x}_{diag3}$ a one-hot encoded vector $\in \{0,1\}^{|V_{diagnosis}|}$ representing the diagnosis category registered for the tertiary diagnosis field
4. $\overline{x}_{countdiag}$ a vector $\in \mathbb{R}^{|V_{diagnosis}|}$ representing the count of diagnosis categories registered in all 25 diagnosis fields

- Procedures: every claim in the dataset includes 15 ordered fields, each registering patient's administered procedure category based on CCS grouper during the corresponding hospitalization event. We first extracted set $V_{procedures}$ representing the top procedures having at least 1000 counts registered in HF dataset. Then, we constructed the following vectors:

1. $\overline{x}_{proc1}$ a one-hot encoded vector $\in \{0,1\}^{|V_{procedures}|}$ representing the procedure category registered for the primary procedure field
2. $\overline{x}_{proc2}$ a one-hot encoded vector $\in \{0,1\}^{|V_{procedures}|}$ representing the procedure category registered for the secondary procedure field
3. $\overline{x}_{proc3}$ a one-hot encoded vector $\in \{0,1\}^{|V_{procedures}|}$ representing the procedure category registered for the tertiary procedure field
4. $\overline{x}_{countproc}$ a vector $\in \mathbb{R}^{|V_{procedures}|}$ representing the count of procedure categories registered in all 15 procedure fields

- Body-system chronic condition: every claim in the dataset includes 25 ordered fields, each representing body-system chronic condition indicators, categorizing ICD-9-CM diagnosis codes into chronic or not [12]. We refer to the list of body-system categories by set $V_{bchronic}$ that includes 18 categories. We constructed the following vectors:

1. $\overline{x}_{bchronic1}$ a one-hot encoded vector $\in \{0,1\}^{|V_{bchronic}|}$ representing the body-system chronic condition indicator category listed in the primary field
2. $\overline{x}_{bchronic2}$ a one-hot encoded vector $\in \{0,1\}^{|V_{bchronic}|}$ representing the body-system chronic condition indicator category listed in the secondary field
3. $\overline{x}_{bchronic3}$ a one-hot encoded vector $\in \{0,1\}^{|V_{bchronic}|}$ representing the body-system chronic condition indicator category listed in the tertiary field
4. $\overline{x}_{countbchronic}$ a vector $\in \mathbb{R}^{|V_{bchronic}|}$ representing the count of body-system chronic condition indicator categories registered in the 25 fields

- External cause of injury code: every claim in the dataset includes 4 ordered fields, each detailing an injury code (E-code) based on CCS software categorizing all ICD-9-CM diagnosis codes into 20 categories [12]. We refer to the list of E-code categories by set V_{ecode} that includes 20 categories. We constructed the following vectors:

1. $\overline{x}_{ecode1}$ a one-hot encoded vector $\in \{0,1\}^{|V_{ecode}|}$ representing the E-code injury category listed in the primary field
2. $\overline{x}_{countecode}$ a vector $\in \mathbb{R}^{|V_{ecode}|}$ representing the count of E-code injury categories registered in the 4 fields

- Procedure classes: every claim in the dataset includes 15 ordered fields, each describing a broad category code (i.e. class) based on categorization of the ICD-9-CM procedure codes [12]. We refer to the list of procedure broad categories by set $V_{procedureclass}$ that includes 4 categories. We constructed the following vectors:

  1. $\overline{x}_{countpclass}$ a vector $\in \mathbb{R}^{|V_{procedureclass}|}$ representing the count of procedure class categories registered in the 15 fields

- Comorbidity condition: every claim in the dataset includes 29 binary fields $\in \{0, 1\}$, each representing an indicator of a specific comorbidity that was determined by the AHRQ comorbidity software [13]. The software determines comorbidities that are more likely present prior to hospitalization event [13]. We refer to the comorbidity categories encoded in the 29 binary variables by set $V_{comorbid}$. We constructed the following vector:

  1. $\overline{x}_{comorbid}$ a vector $\in \{0, 1\}^{|V_{comorbid}|}$ where each component corresponds to one of the 29 binary fields representing the presence/absence of a comorbidity condition

- Major diagnostic category (MDC) assigned by HCFA DRG Grouper algorithm during the processing of HCUP dataset [12]. We refer to the list of MDC categories by set V_{mdc} where we constructed the following vector:

  1. $\overline{x}_{mdc}$ a one-hot encoded vector $\in \{0, 1\}^{|V_{mdc}|}$ representing the MDC code/category

- Risk of mortality subclass: every claim in the dataset includes a field that measures risk of mortality subclass based on all patient refined diagnosis related groups assigned using software developed by 3M Health Information System [12]. The measure consists of five categories which we refer to by the set $V_{riskmortal}$. We constructed the following vector:

  1. $\overline{x}_{riskmortal}$ a one-hot encoded vector $\in \{0, 1\}^{|V_{riskmortal}|}$ representing the risk of mortality subclass category

- Severity of illness subclass: every claim in the dataset includes a field that measures severity of illness subclass based on all patient refined diagnosis related groups assigned using software developed by 3M Health Information System [12]. The measure consists of five categories which we refer to set $V_{severity}$. We constructed the following vector:

  1. $\overline{x}_{severity}$ a one-hot encoded vector $\in \{0, 1\}^{|V_{severity}|}$ representing the severity of illness subclass category

- Major operating room procedure indicator: $x_{orproc} \in \{0, 1\}$ a binary variable indicating whether a major operating room procedure was reported on discharge

- Number of chronic conditions: $x_{nchronic} \in \mathbb{R}$ a variable representing the counts of unique chronic diagnosis reported on the discharge

- Socio-demographics: every claim is associated with a patient and includes information regarding patient's age, gender, income and place/location of residence. We constructed a vector $\overline{x}_{sociodem}$ that represents the concatenation of the following variables:
  1. age: $x_{age} \in \mathbb{R}$ a variable representing the age of the patient
  2. gender: $x_{gender} \in \{0, 1\}$ a binary variable representing the gender of a patient
  3. income: $\overline{x}_{income} \in \{0, 1\}^{|V_{income}|}$ a one-hot encoded vector indicating the median household income quartiles for patient's zip code, where V_{income} is the set of income categories
  4. place/location: $\overline{x}_{ploc} \in \{0, 1\}^{|V_{ploc}|}$ a one-hot encoded vector describing patient's location based on the National Center for Health Statistics (NCHS) classification scheme for US counties, where V_{ploc} is the set of location categories
  5. resident: $x_{resident} \in \{0, 1\}$ a binary variable representing if the patient is resident in the state in which they were treated
- Event/claim info: every claim included the length of stay of the hospitalization event, if the admission was on a weekend, and the discharge month. We also computed the time difference between the hospital admission of a current claim/event and the discharge of previous claim/event for all events in a patient's timeline. We constructed a vector $\overline{x}_{event}$ that is the concatenation of the following variables:
  1. length of stay: $x_{los} \in \mathbb{R}$ a variable representing the length of stay in days for each hospitalization event
  2. time difference between consecutive events: $x_{\Delta t} \in \mathbb{R}$ a variable representing the time difference in days between current admission and previous discharge events
  3. admission on weekend: $x_{aweekend} \in \{0, 1\}$ a binary variable indicating if a patient was admitted on a weekend
  4. discharge month: $\overline{x}_{dmonth} \in \{0, 1\}^{|V_{dmonth}|}$ a one-hot encoded vector indicating a patient's discharge month where V_{dmonth} is the set of recorded months in the dataset
  5. disposition of patient: $\overline{x}_{dispuniform} \in \{0, 1\}^{|V_{dispuniform}|}$ a one-hot encoded vector indicating the disposition of the patient at discharge
  6. expected primary payer: $\overline{x}_{paysrc} \in \{0, 1\}^{|V_{paysrc}|}$ a one-hot encoded vector indicating the expected primary payer (such as Medicare, private insurance, etc.)
  7. same-day event: $\overline{x}_{sameday} \in \{0, 1\}^{|V_{sameday}|}$ a one-hot encoded vector identifying transfers and/or same-day stay collapsed records
  8. elective admission: $x_{elective} \in \{0, 1\}$ a binary variable indicating elective versus non-elective admission
  9. rehab transfer: $x_{rehab} \in \{0, 1\}$ a binary variable indicating if the claim is a combined record involving transfer to rehabilitation, evaluation, or other aftercare

10. number of index events: $x_{countindex} \in \mathbb{R}$ a variable representing the number of index events in the timeline of a patient up to the current admission event (inclusive)
11. number of admission events: $x_{countevents} \in \mathbb{R}$ a variable representing the number of admission events in the timeline of a patient up to the current admission event (inclusive)

Hence, the feature vector $\overline{x}_t$ is the concatenation of all these variables, encoding the characteristics of both the event and its corresponding patient at one time step of the patient's trajectory.

2 Experiments

2.1 Hyperparameters optimization

2.1.1 RNN model

The set of all possible hyperparameters configuration (i.e. choice of values for hyperparameters) for RNN models is reported in Table 1. These hyperparameters controlled the network architecture design that is represented in Figure 2.

2.1.2 RNNSS model

The hyperparameters configurations for RNNSS model is reported in Table 2, which controlled the network architecture design depicted in Figure 2.

2.1.3 RNNCRF model

Similar to RNN models, the set of all possible hyperparameters configuration for models using RNN with CRF is reported in Table 3 along with the network architecture design in Figure 3.

2.1.4 CRF and Neural CRF models

CRF only and Neural CRF models' hyperparameters configuration is reported in Tables 4 and 5 respectively along with the network architecture/design in Figure 4.

Parameter name	Set/range values	Best/optimal value
Embedding layer (Blue block) dimension	$\{0, \lfloor d/2 \rfloor, \lfloor d/3 \rfloor, \lfloor d/4 \rfloor\}$ where d is input dimension	0
RNN layer (Yellow block)		
RNN type	{LSTM, GRU, Vanilla RNN}	Vanilla RNN
Hidden vector dimension D_h	$\{8, 16, 32, 64, 128, 256\}$	16
Number of hidden layers	$\{1, 2, 3\}$	1
Dropout probability $p_{dropout}$	$\{0.15, 0.35, 0.5\}$	0.35
Embedding layer (Orange block)	$\{0, D_h, \lfloor D_h/2 \rfloor, \lfloor D_h/3 \rfloor, \lfloor D_h/4 \rfloor\}$	0
Non-linear function	$\{tanh, ReLU\}$	$ReLU$
l_2-norm regularization λ	$\{10^{-3}, 10^{-2}, 10^{-1}\}$	10^{-2}
Convex combination parameter for the RNN objective function, α	$\{0.65, 0.8, 0.95\}$	0.8
Batch size during training $\lvert B \rvert$	$\{8, 16, 32, 64, 128\}$	64
Optimization algorithm	{Adam}	Adam

Table 1: RNN hyperparameter options (see Figure 2)

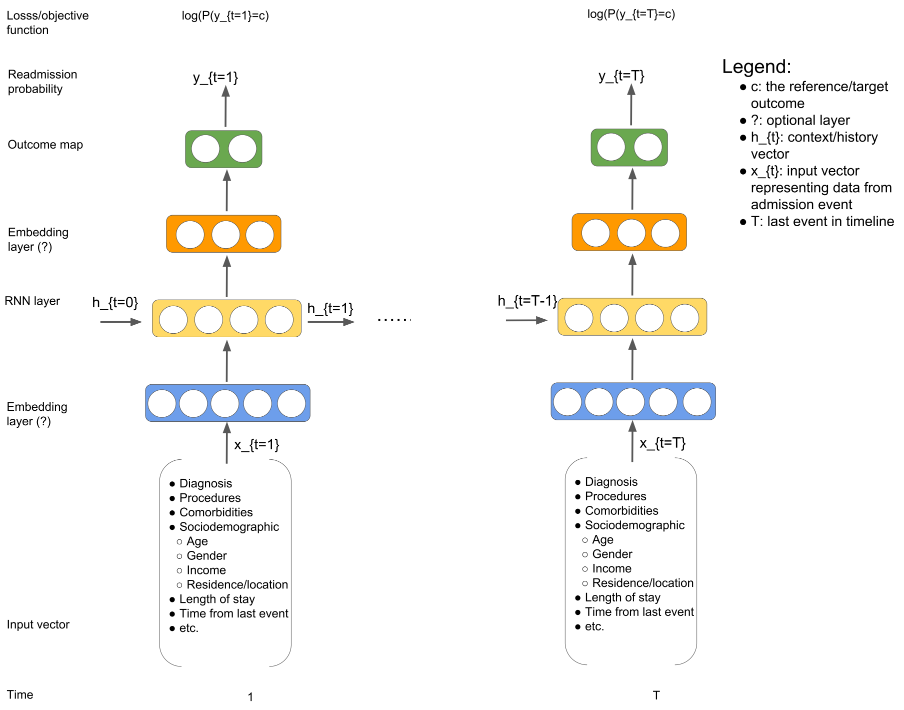

Figure 2: RNN generic model/architecture

Parameter name	Set/range values	Best/optimal value
Embedding layer (Blue block) dimension	$\{0, \lfloor d/2 \rfloor, \lfloor d/3 \rfloor, \lfloor d/4 \rfloor\}$ where d is input dimension	$\lfloor d/2 \rfloor$
RNN layer (Yellow block)		
RNN type	{LSTM, GRU, Vanilla RNN}	GRU
Hidden vector dimension D_h	$\{8, 16, 32, 64, 128, 256\}$	128
Number of hidden layers	$\{1, 2, 3\}$	1
Dropout probability $p_{dropout}$	$\{0.15, 0.35, 0.5\}$	0.15
Embedding layer (Orange block)	$\{0, D_h, \lfloor D_h/2 \rfloor, \lfloor D_h/3 \rfloor, \lfloor D_h/4 \rfloor\}$	$\lfloor D_h/3 \rfloor$
Non-linear function	$\{tanh, ReLU\}$	$tanh$
l_2-norm regularization λ	$\{10^{-3}, 10^{-2}, 10^{-1}\}$	10^{-2}
Convex combination parameter for the RNN objective function, α	$\{0.65, 0.8, 0.95\}$	0.8
Batch size during training $\|B\|$	$\{8, 16, 32, 64, 128\}$	64
Scheduled sampling parameter ρ	{Linear, Exponential, Sigmoid}	Exponential
Optimization algorithm	{Adam}	Adam

Table 2: RNNSS hyperparameter options (see Figure 2)

Parameter name	Set/range values	Best/optimal value
Embedding layer (Blue block) dimension	$\{0, \lfloor d/2 \rfloor, \lfloor d/3 \rfloor, \lfloor d/4 \rfloor\}$ where d is input dimension	$\lfloor d/2 \rfloor$
RNN layer (Yellow block)		
RNN type	{LSTM, GRU, Vanilla RNN}	GRU
Hidden vector dimension D_h	$\{8, 16, 32, 64, 128, 256\}$	128
Number of hidden layers	$\{1, 2, 3\}$	1
Dropout probability $p_{dropout}$	$\{0.15, 0.35, 0.5\}$	0.15
Embedding layer (Orange block)	$\{0, D_h, \lfloor D_h/2 \rfloor, \lfloor D_h/3 \rfloor, \lfloor D_h/4 \rfloor\}$	$\lfloor D_h/3 \rfloor$
Non-linear function	$\{tanh, ReLU\}$	$tanh$
l_2-norm regularization λ	$\{10^{-3}, 10^{-2}, 10^{-1}\}$	10^{-2}
Batch size during training $\|B\|$	$\{8, 16, 32, 64, 128\}$	64
Optimization algorithm	{Adam}	Adam

Table 3: RNNCRF hyperparameter options (see Figure 3)

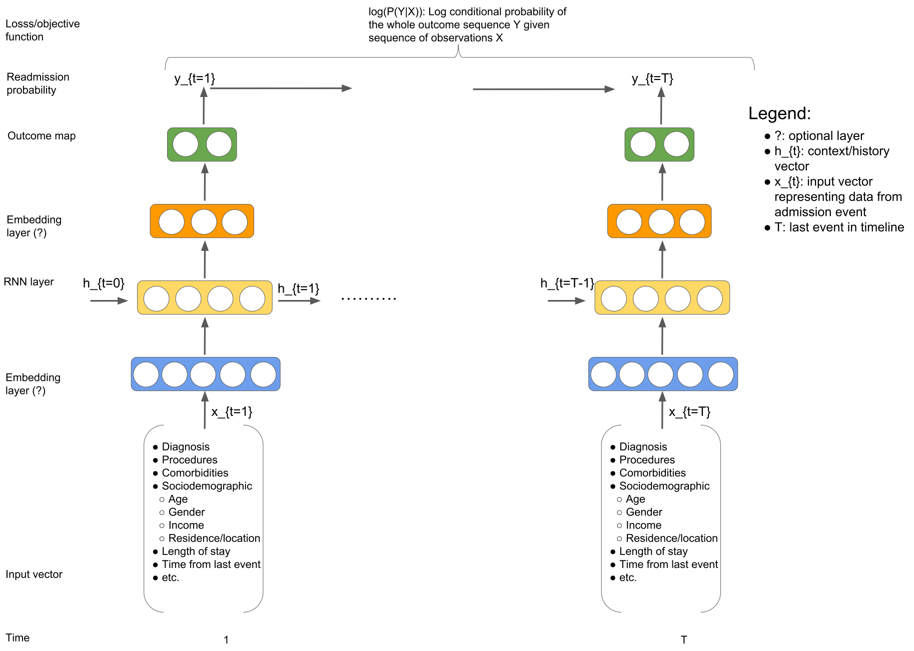

Figure 3: RNNCRF generic model/architecture

Parameter name	Set/range values	Best/optimal value
l_2-norm regularization λ	$\{10^{-3}, 10^{-2}, 10^{-1}\}$	10^{-2}
Batch size during training $\|B\|$	$\{8, 16, 32, 64, 128\}$	64
Optimization algorithm	{Adam}	Adam

Table 4: CRF hyperparameter options (see Figure 4)

Parameter name	Set/range values	Best/optimal value
Embedding layer (Blue block) dimension	$\{0, \lfloor d/2 \rfloor, \lfloor d/3 \rfloor, \lfloor d/4 \rfloor\}$ where d is input dimension	$\lfloor d/2 \rfloor$
Dropout probability $p_{dropout}$	$\{0.15, 0.35, 0.5\}$	0.15
Embedding layer (Orange block)	$\{0, \lfloor D_l/2 \rfloor, \lfloor D_l/3 \rfloor, \lfloor D_l/4 \rfloor\}$ where D_l is input dimension from previous layer l	$\lfloor D_l/3 \rfloor$
Non-linear function	$\{tanh, ReLU\}$	$tanh$
l_2-norm regularization λ	$\{10^{-3}, 10^{-2}, 10^{-1}\}$	10^{-2}
Batch size during training $\|B\|$	$\{8, 16, 32, 64, 128\}$	64
Optimization algorithm	{Adam}	Adam

Table 5: Neural CRF hyperparameter options (see Figure 4)

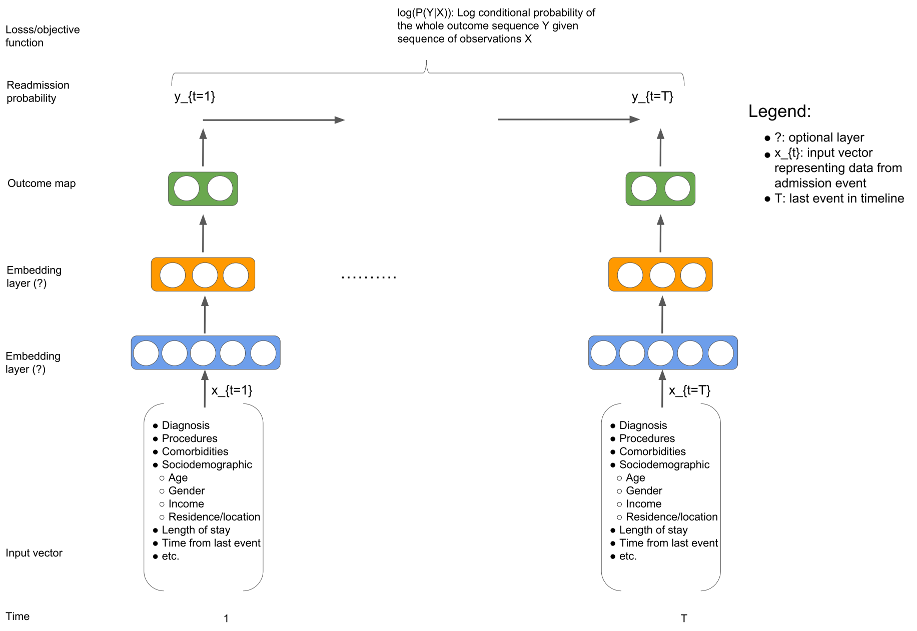

Figure 4: CRF generic model/architecture

2.1.5 CNN model

CNN model's hyperparameters configuration is reported in List 1 along with the network architecture/design in Figure 5.

2.1.6 CNN-Wide model

CNN-Wide model's hyperparameters configuration is reported in List 2 along with the network architecture/design in Figure 6.

2.1.7 MLP model

MLP model's hyperparameters configuration is reported in List 3 along with the network architecture/design in Figure 7.

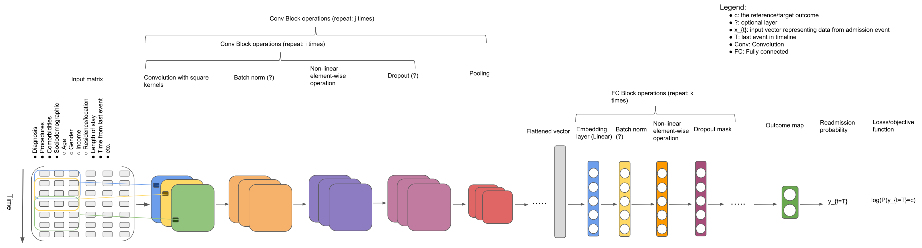

Figure 5: CNN generic model/architecture

List 1 CNN hyperparameter options (see Figure 5). Best parameters are colored in blue.

CNN hyperparameters configuration

- Conv Block operations
  - Conv Block J
    - Conv Block I
      - Square kernel size .... $\{3 \times 3, 5 \times 5\}$
      - Batch norm .... $\{True, False\}$
      - Non-linear function .... $\{tanh, ReLU\}$
      - Dropout .... $\{0, 0.15\}$
      - Starting number of kernels .... $\{64, 128, 256\}$
      - Number of repeats for Conv Block I .... $\{1, 2, 3\}$
    - Pooling .... $\{AvgPool, MaxPool\}$
    - Number of repeats for Conv Block J .... $\{7, 8\}$
- FC Block operations
  - Embedding layer dimension .... $\{\lfloor D_l/3 \rfloor, \lfloor D_l/4 \rfloor\ \lfloor D_l/5 \rfloor\}$ where D_l is the dimension of flattened feature vector from the last convolutional layer
  - Batch norm .... $\{True, False\}$
  - Non-linear function .... $\{tanh, ReLU\}$
  - Dropout .... $\{0, 0.15, 0.35, 0.5\}$
  - Number of repeats for FC Block K .... $\{1, 2\}$
- l_2-norm regularization λ .... $\{10^{-3}, 10^{-2}, 10^{-1}\}$
- Batch size during training $|B|$ .... $\{8, 16, 32\}$
- Optimization algorithm .... $\{Adam\}$

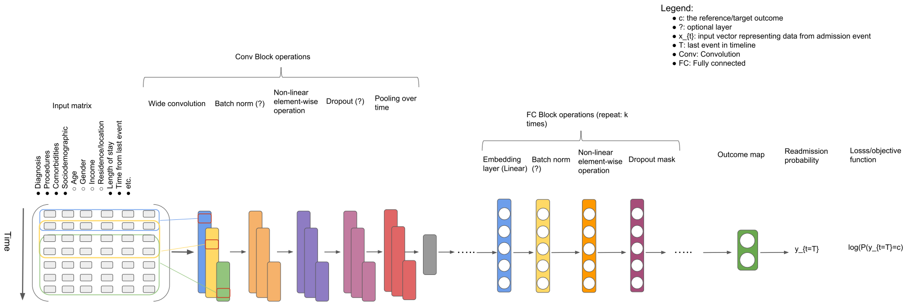

Figure 6: CNN generic model/architecture

List 2 CNNWide hyperparameter options (see Figure 6). Best parameters are colored in blue.

CNN-Wide hyperparameters configuration
- Conv Block operations
  - Rectangular kernel size ..... $\{2\times d, 3\times d, 5\times d\}$ where d is the input dimension
  - Batch norm ..... $\{True, False\}$
  - Non-linear function ..... $\{tanh, ReLU\}$
  - Dropout ..... $\{0, 0.15\}$
  - Number of kernels ..... $\{16, 32, 64, 128\}$
  - Apply padding ..... $\{True, False\}$
  - Pooling ..... $\{AvgPool, MaxPool\}$
  - Number of kernel types used ..... $\{2, 3\}$
- FC Block operations
  - Embedding layer dimension ..... $\{D_l, \lfloor D_l/2\rfloor, \lfloor D_l/3\rfloor, \lfloor D_l/4\rfloor\}$ where D_l is the dimension of flattened feature vector from the last convolutional layer
  - Batch norm ..... $\{True, False\}$
  - Non-linear function ..... $\{tanh, ReLU\}$
  - Dropout ..... $\{0, 0.15, 0.35, 0.5\}$
  - Number of repeats for FC Block K ..... $\{1, 2\}$
- l_2-norm regularization λ ..... $\{10^{-3}, 10^{-2}, 10^{-1}\}$
- Batch size during training $|B|$ ..... $\{8, 16, 32\}$
- Optimization algorithm ..... $\{Adam\}$

List 3 NN hyperparameter options (see Figure 7). Best parameters are colored in blue.

NN hyperparameters configuration
- FC Block operations
  - Embedding layer dimension ..... $\{\lfloor D_l/2\rfloor, \lfloor D_l/3\rfloor, \lfloor D_l/4\rfloor\}$ where D_l is the dimension of feature vector from the previous layer
  - Batch norm ..... $\{True, False\}$
  - Non-linear function ..... $\{tanh, ReLU\}$
  - Dropout ..... $\{0, 0.15, 0.35, 0.5\}$
  - Number of repeats for FC Block K ..... $\{1, 2, 3, 4, 5\}$
- l_2-norm regularization λ ..... $\{10^{-3}, 10^{-2}, 10^{-1}\}$
- Batch size during training $|B|$ ..... $\{32, 64, 128\}$
- Optimization algorithm ..... $\{Adam\}$

Parameter name	Set/range values	Best/optimal value
l_1-norm regularization λ	$\{10^{-3}, 10^{-2}, 10^{-1}\}$	10^{-1}
Optimization algorithm	{Liblinear, Saga}	Liblinear/Saga
Weighting scheme (i.e. weighting training examples)	{Balanced, None}	Balanced

Table 6: Logistic regression with l_1-norm regularization (LASSO)

Parameter name	Set/range values	Best/optimal value
l_2/l_1-norm regularization λ	$\{10^{-3}, 10^{-2}, 10^{-1}, 1\}$	10^{-1}
Optimization algorithm	{Liblinear, Saga}	Liblinear/Saga
Weighting scheme (i.e. weighting training examples)	{Balanced, None}	Balanced

Table 7: Logistic regression with l_2-norm regularization

2.1.8 Logistic regression

Logistic regression models' hyperparameters options are reported in Tables 6 and 7 respectively.

2.2 Feature importance

2.2.1 Logistic regression

The analysis of feature importance is reported in Figure 8, which shows the normalized coefficients of the trained LASSO models averaged across all folds.

2.2.2 RNNCRF model

For the best neural model (RNNCRF), we report the analysis of feature importance according to an approach previously reported in [14]. In short, we iterated over all features attached to the last HF event, and computed the probability of readmission with a feature present or absent. The difference between both probabilities allowed us to quantify a feature's importance across the five folds (we call this metric *diff_prob* see Fig. 9). Additionally, we computed another variation of the same metric by incorporating the percentage of occurrence of each feature (i.e. when the feature is present) in the computation. In other words, we weighted the computed differences by the percentage of time each feature was present in the dataset (referred to *diff_prob_weighted*, see Fig. 10). A third variation, is computing a ratio (for every feature) dividing the average difference in probability (*diff_prob*) by the average value of the feature when it was present and again weighted by the percentage of occurrence of the feature (*ratio_diff_prob_weighted*, see Fig. 11).

2.3 Models' performance across five-folds

Figure 12 reports the ROC and average ROC curves of the best models across all five-folds.

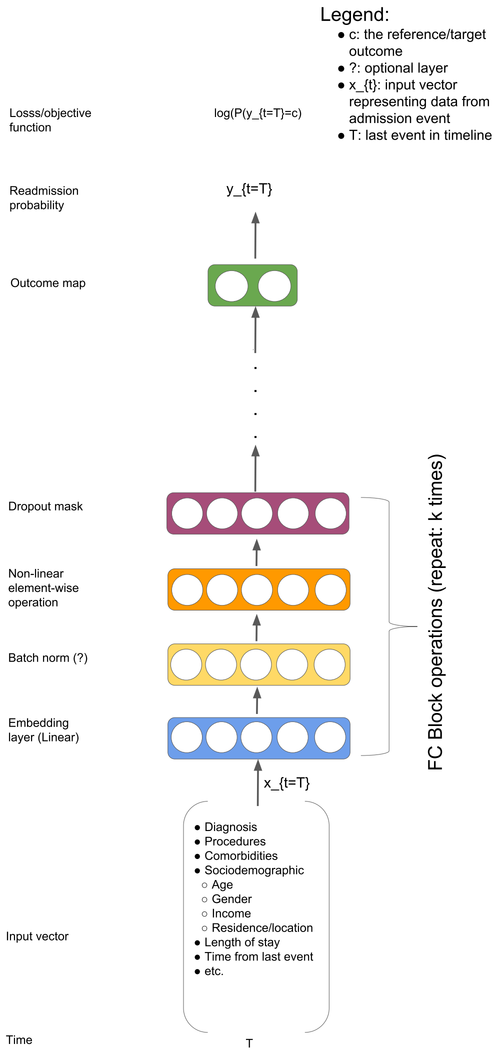

Figure 7: NN generic model/architecture

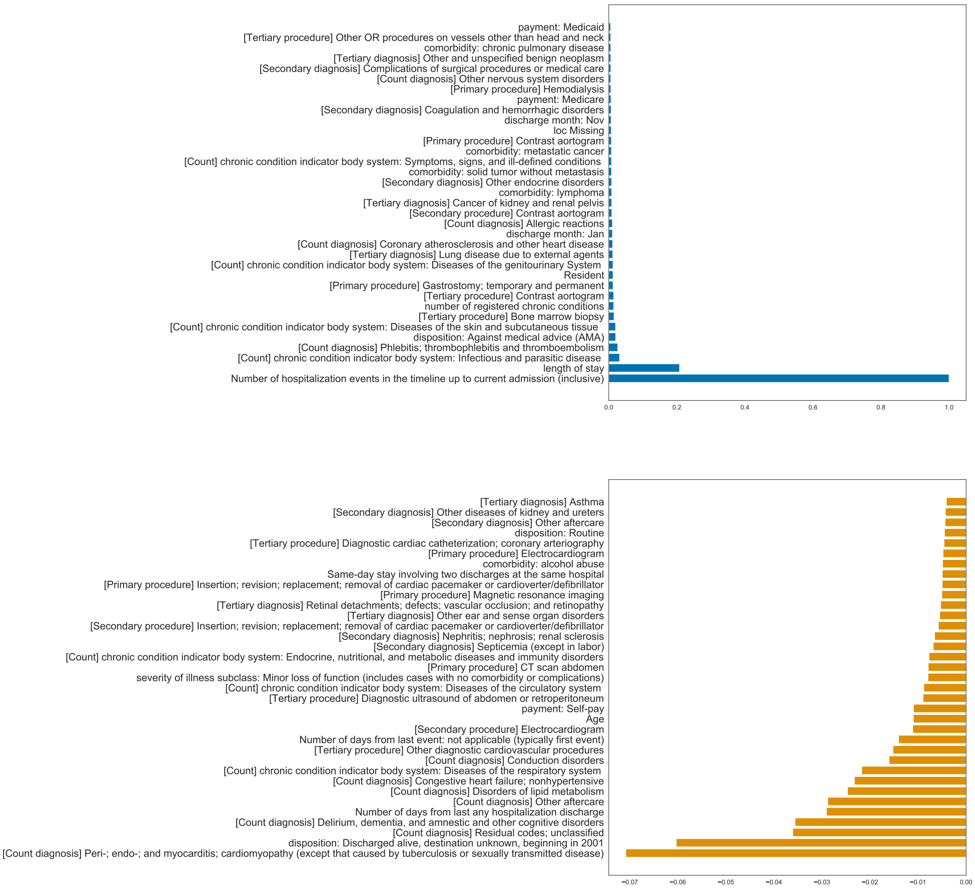

Figure 8: Top-35 features (normalized) in LASSO models contributing to the increase and decrease of log-odds of readmission

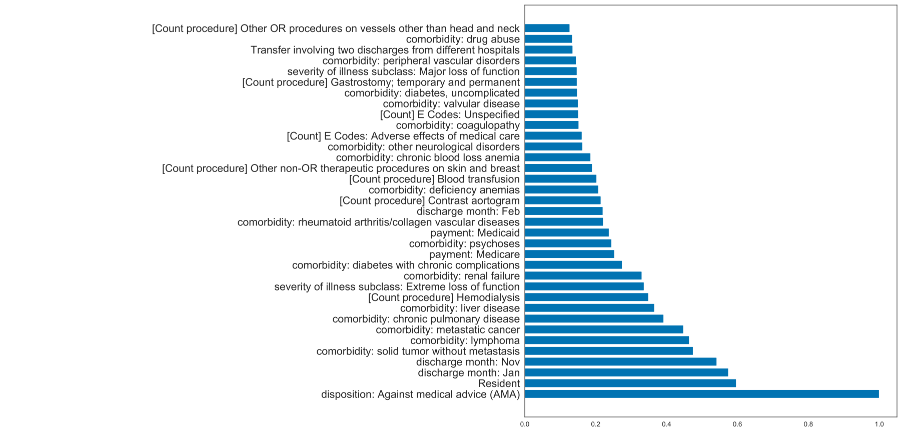

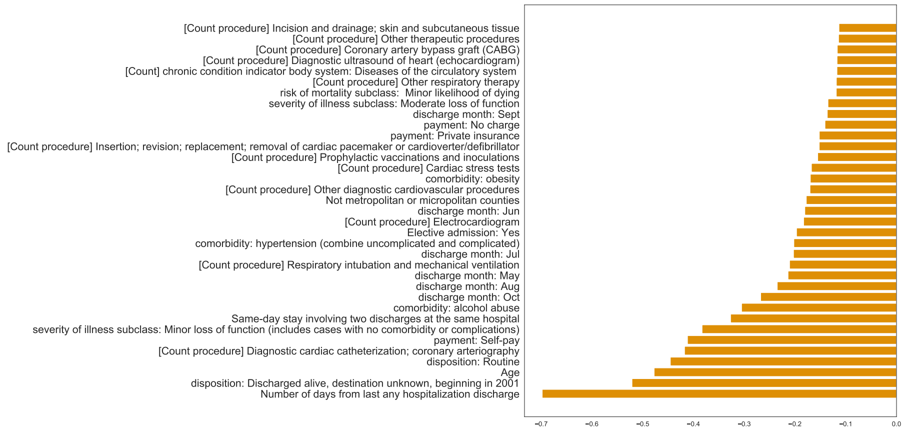

Figure 9: Top-35 features (normalized) in RNNCRF models using *diff_prob* metric

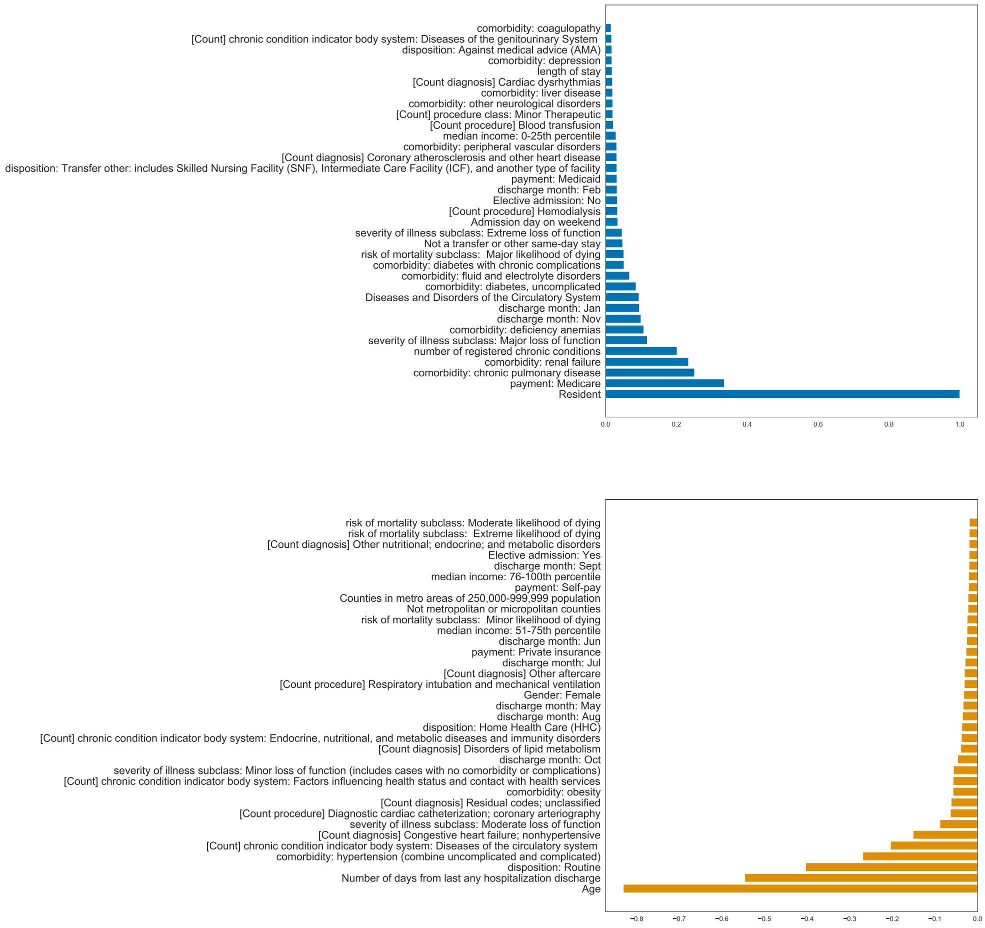

Figure 10: Top-35 features (normalized) in RNNCRF models using *diff_ prob_ weighted* metric

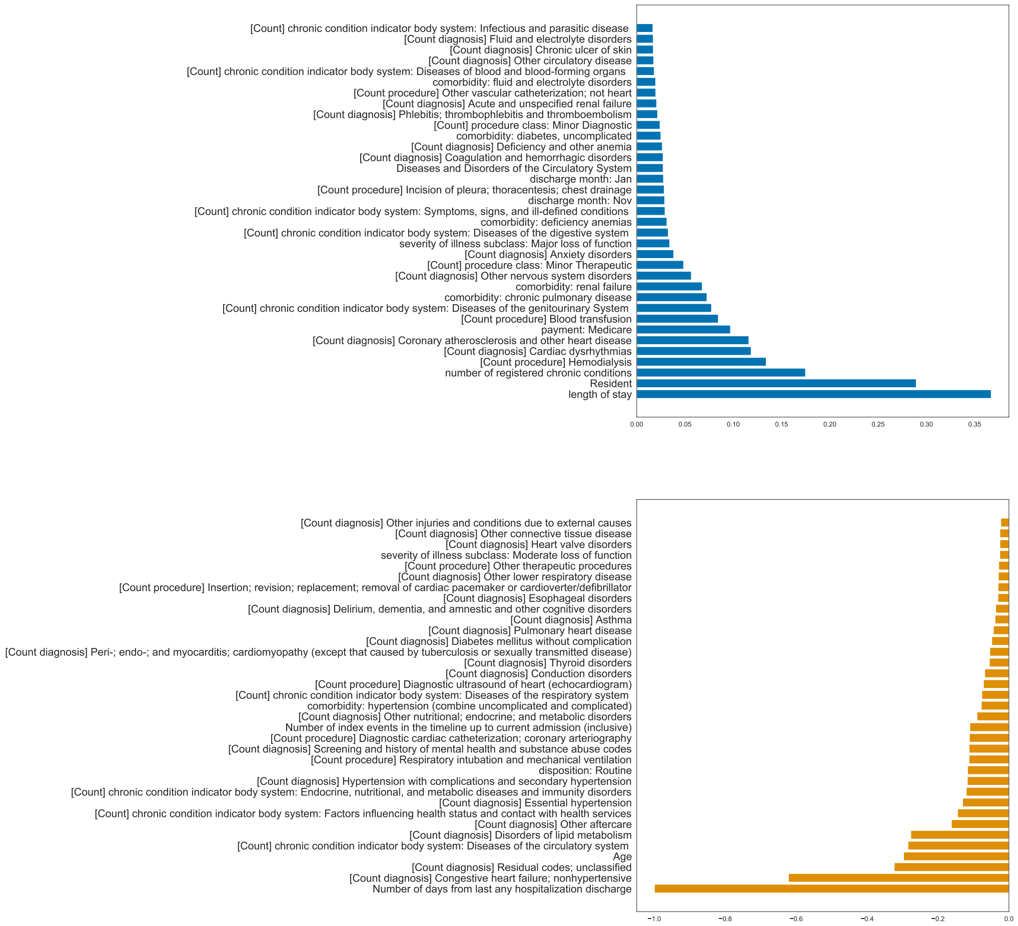

Figure 11: Top-35 features (normalized) in RNNCRF models using *ratio_diff_prob_weighted* metric

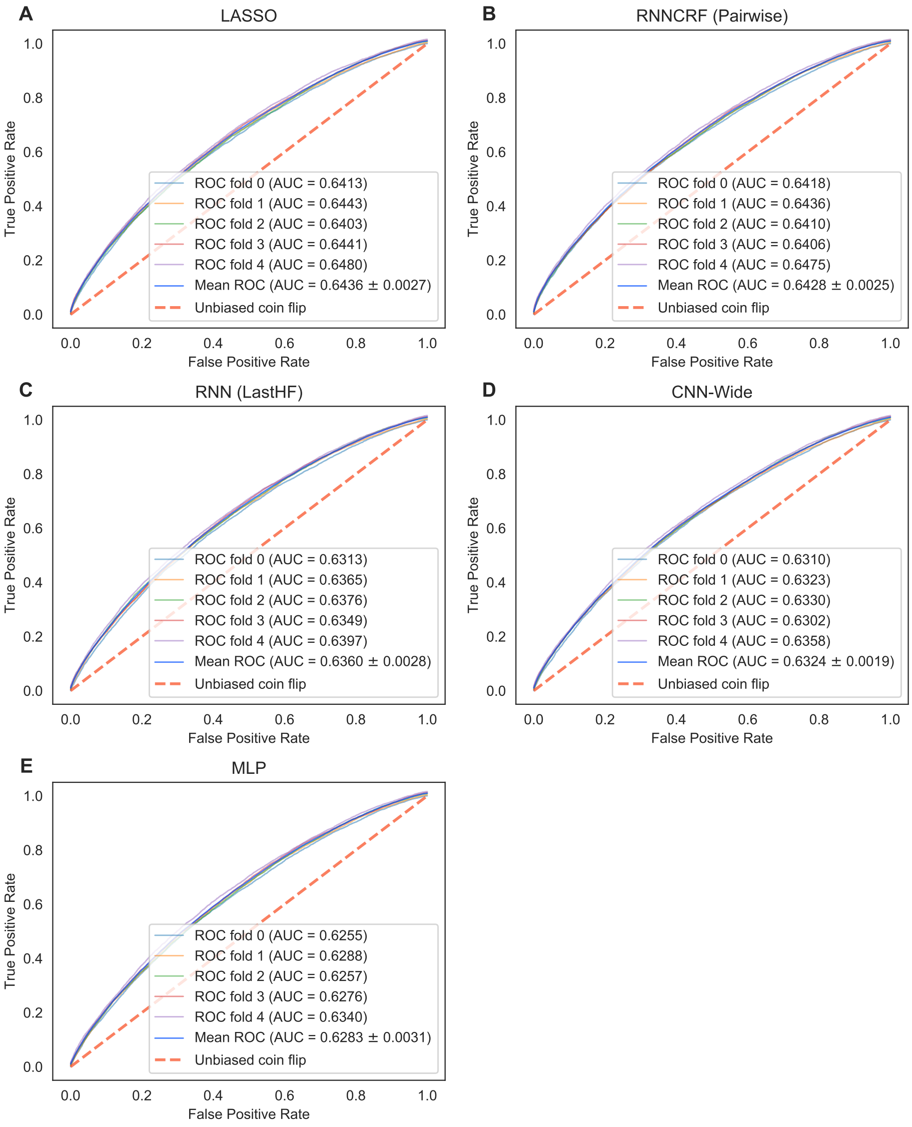

Figure 12: ROC curves for the best models across all five-folds

3 References

[1] Hochreiter, S. & Schmidhuber, J. Long Short-Term Memory. *Neural Computation* **9**, 1735–1780 (1997).

[2] Graves, A. *Supervised Sequence Labelling with Recurrent Neural Networks*, vol. 385 of *Studies in Computational Intelligence* (Springer Berlin Heidelberg, Berlin, Heidelberg, 2012).

[3] Cho, K. *et al.* Learning Phrase Representations using RNN Encoder-Decoder for Statistical Machine Translation. In *Proceedings of the 2014 Conference on Empirical Methods in Natural Language Processing (EMNLP)*, 1724–1734 (Association for Computational Linguistics, Doha, Qatar, 2014).

[4] Lipton, Z. C., Kale, D. C., Elkan, C. & Wetzel, R. Learning to Diagnose with LSTM Recurrent Neural Networks (2015). 1511.03677.

[5] Collins, M. Log-Linear Models, MEMMs, and CRFs.

[6] Ye, N., Lee, W. S., Chieu, H. L. & Wu, D. Conditional Random Fields with High-Order Features for Sequence Labeling. *Neural Information Processing Systems* **2**, 2 (2009).

[7] Cuong, N. V., Ye, N., Lee, W. S. & Chieu, H. L. Conditional Random Field with High-order Dependencies for Sequence Labeling and Segmentation. *Journal of Machine Learning Research* **15**, 981–1009 (2014).

[8] Vieira, T., Cotterell, R. & Eisner, J. Speed-Accuracy Tradeoffs in Tagging with Variable-Order CRFs and Structured Sparsity. In *Emnlp*, 1973–1978 (Association for Computational Linguistics, Austin, Texas, 2016).

[9] Allam, A. & Krauthammer, M. PySeqLab: an open source Python package for sequence labeling and segmentation. *Bioinformatics* **33**, 3497–3499 (2017).

[10] Ma, X. & Hovy, E. End-to-end Sequence Labeling via Bi-directional LSTM-CNNs-CRF. In *Proceedings of the 54th Annual Meeting of the Association for Computational Linguistics*, 1064–1074 (Association for Computational Linguistic, Berlin, Germany, 2016).

[11] HCUP Software. HCUP Clinical Classifications Software (CCS) for ICD-9-CM (2009).

[12] HCUP Software. HCUP Chronic Condition Indicator (CCI) (2009).

[13] HCUP Software. HCUP Comorbidity Software (2008).

[14] Avati, A. *et al.* Improving palliative care with deep learning. In *2017 IEEE International Conference on Bioinformatics and Biomedicine (BIBM)*, 311–316 (IEEE, 2017).
